# Family planning and abortion service availability and utilisation during the COVID-19 pandemic in Ghana

**DOI:** 10.1186/s12978-025-02122-x

**Published:** 2025-11-20

**Authors:** Deda Ogum, Ernest Tei Maya, Emefa Modey, Adom Manu, Kwasi Torpey

**Affiliations:** School of Public Health, University of Ghana, Legon Accra, Ghana

**Keywords:** Family planning, Sexual & Reproductive Health services, Ghana, Abortion, COVID-19, Health systems, Pandemic

## Abstract

**Background:**

The effect of COVID-19 has manifested both in the capacity of healthcare systems to provide services as well as create a good balance between pandemic management and maintenance of essential health services. Earlier studies in Ghana during the pandemic reported low patronage of family planning (FP) services but a sudden spike in emergency contraceptive pill utilization. This paper seeks to assess health service availability and readiness, client needs for, and utilization of FP and abortion services during the COVID-19 pandemic period in Ghana.

**Methods:**

This study was a panel study with two-time data collection points six to nine months apart. Both quantitative and qualitative approaches were used. A one-time survey was used to assess SRH service utilization by 997 clients. Qualitative data involved a total of 24 Focus Group Discussions (FGDs), 128 In-depth Interviews (IDIs) with female clients and their male partners, and 32 IDIs with healthcare practitioners in the four selected facilities. Also, the WHO Service Availability, Readiness and Assessment tool was completed for the health facilities at baseline and endline. Descriptive statistics and thematic analysis were conducted for quantitative and qualitative data respectively.

**Results:**

Age of clients and their male partners participating in IDIs ranged between 18 and 50 years (mean = 33.2 years) while participants for community FGDs ranged between 16 and 56 years (mean = 32.0 years).

The majority (68%) of clients visiting the health facility for SRH care sought FP services while 5% sought abortion/post-abortion care of which 71% needed post-abortion care. Attendance data showed sensitivity to the occurrence of the different waves of COVID-19. Family planning and abortion services were generally available but witnessed some short-lived disruption. Healthcare managers reported financial stress which led to innovations in procurement of PPEs and hand sanitizers. Telemedicine facilities did not provide SRH care. Fear of stigma was a major barrier to access to abortion care.

**Conclusion:**

The relatively low COVID-19 infection rates in Ghana preceded by the national COVID-19 preparedness strategy may explain the low impact on disruption of FP and abortion services. Development of SRH specific guidelines and strengthening telemedicine facilities to include SRH care may reduce future disruption.

**Supplementary Information:**

The online version contains supplementary material available at 10.1186/s12978-025-02122-x.

## Abstract (plain language summary)

Since 2020 most countries around the world were hit by COVID-19 infection with many deaths recorded. Measures to control the pandemic while sustaining existing services stressed health systems. Earlier studies in Ghana during the pandemic reported impact on health services. Yet, we do not know how the COVID-19 pandemic impacted the health system’s readiness and availability to provide family planning and abortion services, and client utilization, at different levels of care in public health facilities in Ghana. This study involved a survey of clients accessing care at all sexual and reproductive health units in the 4 selected hospitals. We also had in-depth interviews with 128 women and their male partners, 32 interviews with healthcare providers and 24 groups of community members to elicit their needs for sexual and reproductive health services, utilization, and perspectives on care provided at the time.

We found that there was some disruption of family planning and abortion services, especially for surgical abortion methods which were suspended during the lockdown and period thereafter. However, service provision was fully restored in all facilities by the follow-up period. Fear of COVID-19 infection and stigma were barriers to accessing care even after lockdown was ended. We recorded innovative measures such as self-production of personal protective equipment and institution of telemedicine facilities. Client utilization data showed patterns related to waves of infection in Ghana. Thus, we believe that the minimal disruptions observed was due to the relatively low infection rates in Ghana.

**Supplementary Information:**

The online version contains supplementary material available at 10.1186/s12978-025-02122-x.

## Background/Introduction

The global COVID-19 pandemic has had significant impact on health service provision in all global regions. The impact has manifested both in the capacity of the healthcare system to provide services as well as create a good balance between managing the pandemic itself and maintaining essential health services, thus, exposing the inefficiencies of healthcare systems and widening access gaps across populations [1, 2].

Actions to contain pandemics often led to the suspension of many routine and elective services that were otherwise classified as essential, including sexual and reproductive health. The typical effect of pandemics such as service and supply chain disruptions, challenges with health workforce, policy changes, community and individual barriers and shifts in advocacy often spells negative SRH outcomes for the population. Lessons learned from earlier pandemic situations show an increase in the number of unwanted pregnancies, unsafe abortions, maternal deaths, increase in STIs following even the slightest disruption of SRH services and supply of commodities. Similarly, there is evidence that the COVID-19 pandemic has limited access to SRH services around the world [3]. This obviously has negative impact on female health and wellbeing stifling global efforts at ensuring gender equality and SRH rights [4].

Reports show that the challenges which led to a reduction in utilisation of services at the peak of the pandemic were not only limited to individual barriers such as fear of infection or financial difficulty, nor health facility barriers such as shortage of commodities, but was most influenced by a lack of information about the continuity of SRH services [5]. Another major influence reported at the health facility level was the task shifting of SRH staff to support COVID-19 treatment and response, especially in treatment centers and higher-level facilities. In Ghana, Strategies to continue family planning services during the COVID-19 pandemic such as the use of telemedicine and intensified advocacy were proposed to maintain family planning as a priority in the emergency response to COVID-19 [6].

A rapid assessment by the WHO on the continuity of services during COVID-19 from February to May 2020 in 17 countries including Ghana showed that 80% of countries had integrated SRH services with all its components into their systems (5). Evidence from regular WHO assessment on SRH services had documented low patronage of family planning services amidst stockout of commodities and largely attributable to restrictions on movement(5). However, there was evidence to show continuous service delivery across the globe and a rapid reversal of this trend after lockdown [7].

Evidence from Ghana indicate unequal disruptions in essential health services during the COVID-19 pandemic in settings with high COVID-19 cases. Data from the Ghana Health Service District Health Management Information System II(DHMIS II) showed a 25–65% decline in maternity health service utilization in a referral facility [8]. Earlier studies in Ghana during the pandemic reported low patronage of family planning services during the pandemic but a sudden spike in the utilization of emergency contraceptive pill (ECP), and indication of minimal disruption in use of ECPs during the period [9, 10]. Other studies report that where other modern contraceptives, such as male condoms, were reportedly used regularly, they were obtained prior to the lockdown [9]. Data from the Ghana Health Service District Health Management Information System II(DHMIS II) from 2019 to 2023 show that, family planning acceptor rates reduced by 25% from 2019 to 2020. Whilst a further 15% reduction was observed in 2021, the post pandemic period recorded gradual increases in acceptor rates of 8% in 2022 and 12% in 2022 [11]. The use of traditional and folkloric methods though reportedly used, were accompanied with associated cases of sexually transmitted infections [9]. There is limited evidence on the effect of the pandemic on utilization of SRH services in Ghana and how these changed with the easing of restrictions and subsequent waves of the infection across different levels of the health system. This paper seeks to assess health service availability and readiness, client needs for, and utilization of family planning and abortion services during the COVID-19 pandemic period in Ghana. It interrogates client and health provider perceptions of drivers and barriers to service availability and readiness, and client utilization of family planning and abortion services during lock-down and the period thereafter. It further examines measures taken by the health services to both ensure safety and maintain family planning and abortion service provision during the pandemic.

## Methods

### Study design

This study was a longitudinal study (panel survey) with two data collection time points. The study used both quantitative and qualitative approaches to data collection.

The quantitative aspects involved a one-time survey of clients accessing various SRH services in the participating health facilities as well as assessment of the physical presence of facilities, infrastructure, logistics and readiness to provide various SRH services using the WHO Service Availability and Readiness Assessment (SARA) tool. Additional file 1 shows the data form that was used.

The qualitative aspects of the study used client, provider, and community member interviews to capture perspectives on SRH service availability, readiness and barriers, changes in SRH service needs, service provision and utilization of services. The individual interviews and focus group discussions explored both client and provider perceptions on the impact of the COVID-19. Also, the health care provider interviews used the WHO six building blocks framework with a focus on delivery of contraceptive and abortion care. Further details of methods used in this study are reported elsewhere [12].

### Study area and setting

This study was conducted in four districts in the Greater Accra region: Accra metropolis, Ga-west municipal district, Shai-Osudoku district and the Ashaiman Municipal districts. The Greater Accra region is home to the largest city in Ghana, Accra, which was also the epicenter for the COVID-19 infections in the country. As of April 12 2021, the Greater Accra accounted for 50,527 of the country’s 91,545 confirmed cases.. The average recovery rate in Ghana as of April 2021 was 98%.

The facilities selected includes one that was designated as the national level COVID-19 treatment center, which originally served as the regional referral hospital, two district hospitals that serve as secondary level referral centers in their districts (Shai-Osudoku and Ga-West Municipal) and one primary level facility (Ashaiman polyclinic) which offers essential health care services, including adolescent SRH services to its very youthful, highly populated low-income urban population.

### Study population

Key staff and clients accessing services in the selected facilities, as well as male partners of clients and community members residing within the catchment area of the health facilities were targeted for this study.

The inclusion criteria for clients and their male partners were: (1) Being of reproductive age (15–49 years), (2) They or their partner has utilized or sought to utilize any of the SRH services in the selected facilities (3) willingness to participate in the study with a signed informed consent. Inclusion criteria for community members was residence within the catchment area of selected health facilities and their willingness to participate in study with a signed informed consent form.

The inclusion criteria for selection of healthcare providers (HCP) are: (1) knowledgeable about SRH services provided in the facility; and (2) agree to an in-depth interview regarding SRH service provision with a signed informed consent.

### Sample size, recruitment and sampling

The four facilities involved in the current study were selected based on the following criteria: COVID-19 management status, availability of SRH services, client utilization data, facility’s level of service provision, patient and catchment area characteristics, and facility approval to participate in the study. National and sub-national COVID-19 data was not disaggregated at the district level; hence, it could not be factored into the facility selection. The four facilities selected allowed for a good representation of rural–urban populations, different socio-economic status of clients, health facility level, range of SRH services offered and utilized and critical groups such as adolescents. Considering that the selected facilities have high attendance to SRH services, and the descriptive nature of the client survey, no formal sample size estimation was determined for this study. Thus, the minimum number based on average monthly client service utilisation survey was 961 (See Table 1 below), which is much higher than the minimum of 400 considered adequate for cross-sectional surveys (13). The number interviewed per health facility was sampled proportional to size.

**Table 1:** Characteristics of selected health facilities

Facility	Level of facility^a^	Setting	Monthly attendance		
			Family Planning	STI care	Abortion/PAC
Ga-West Municipal Hospital	2nd Municipal Hospital	Peri-urban/rural	71	20	4
Shai-Osudoku District Hospital	2nd District Hospital	Peri-Urban/rural	145	No information	21^b^
Greater Accra Regional Hospital	3rd Regional hospital	Urban	630^a^	No records	N/A
Ashaiman Polyclinic	1 st Polyclinic	Urban/Slum	64^d^	26^c^	No information

^a^minimum monthly attendance recorded 2020; ^b^total for 2020; ^c^based on 77 cases recorded from Jan to March 2021; ^d^no information available. Estimated at 10% lower than Ga West

#### Quantitative data

Participants for the client survey were consecutively sampled while assessing any of the SRH services available in the selected health facility. Research assistants were well positioned at the various units and interviewed clients upon referral by the focal person in the facility.

Unit heads and hospital in-charges were purposefully sampled to provide details on client utilization of SRH services at baseline (6 months prior to the study) and at endline (next 6–9 months data).

### Qualitative study

Purposive sampling was used to select key health facility staff, clients and their male partners to participate in in-depth interviews (IDIs) and focus group discussions (FGDs). Selection for IDI and FGDs were done separately by the focal persons at the health facility and participants did not have to participate in both.

The study planned to select from each facility approximately 10 to 15 women (until saturation) of reproductive age seeking SRH services; about 6 to 12 men who are partners of women utilizing SRH services and about 4 key health care providers. Table 2 below shows the planned and actual sample sizes used by the study.

**Table 2:** Qualitative study sample sizes

		**HCPs**	**Clients (IDIs)**	**Partners (IDIs)**	**FGD women**	**FGD Men**
**SITE**	Planned (T1/T2)	16	40	40	10	5
	Actual baseline (T1)	16	36	28	9	3
	Actual Endline (T2)	16	36	30	9	3
	Total completed	32	72	58	18	6

### Study measures

#### Service availability & readiness

This refers both to the presence of the health facility and reach to services as reported by the clients. From the provider perspective, this was measured as “unit opened and staff ready to provide specific service(s)”. For clients, this was measured as the evidence/knowledge/perception that the “unit was opened, and staff present to provide specific services”.

#### Access to services

This was assessed qualitatively among clients and their partners. Interview questions elicited information on how they accessed the services and any barriers they may have encountered while trying to or during the service.

#### Needs for SRH service

NThis was assessed during qualitative interviews. “Need for SRH services” was broadly used but specific questions solicited details on client need for contraception and comprehensive abortion care (CAC) services during the COVID-19 period.

#### Utilisation

Utilisation of FP and abortion services were determined based on health facility records and in qualitative interviews. These were captured as the number of clients that used each service 6 months prior to baseline and endline data collection times. The data also included referrals to and from the health facility for FP or abortion services.

#### Acceptability of services

This refers to the sociocultural dimension of the care received as reported by the clients as “acceptable” or “unacceptable’. This was mainly qualitatively assessed.

### Data management and statistical analysis

The quantitative data in this study is meant to provide a basic set of information about the characteristics of the health facilities included. Quantitative data obtained from the one-time client utilization survey as well as client utilization/attendance to health facility for FP and abortion services over the duration of the study were entered into Open Clinica software. The data was imported into STATA for statistical analysis. Descriptive statistics such as frequencies, mean, median and range were determined for study outcomes. Charts, figures and tables were used to display results as appropriate. The aim is to use tracer items for the provisions of FP and abortion services such as availability of guidelines, commodities and staff. These include basic characteristics of the four facilities, the monthly number of clients, types of procedures provided, number of medical staff, stocks of drugs, etc.

Data obtained from qualitative interviews were transcribed verbatim and double coded by two researchers guided by the general questions, specific questions and comparisons with readiness for unexpected emergent results. Both inductive and deductive coding approaches was used by the researchers and analysed thematically.

Integration of analyses of HCP and client/partner datasets: The study sought from the outset to integrate findings from all data collection approaches and sources. The health facility data was compared with the narratives from the qualitative interviews to elicit a more complete understanding of each data source. We planned to dwell more on the qualitative data collected to inform our decisions.

## Results

### Sample description

A total of 990 complete responses were obtained from the client survey. Respondents were age 15 to 47 years, predominantly female (99.5%) and had between zero and 19 years of formal education. The majority (65.3%) of respondents were cohabiting with their partners. A little over a tenth of the respondents tested for COVID-19 and about 7% were pregnant at the time of the survey. More details on survey clients characteristics are shown in Additional file 2.

For the qualitative aspects of this study, a total of 16 healthcare workers interviewed consisted of 3 males and 13 females, all of which were married and Christian, and in management positions in the health facility. Four of them were at the rank of medical superintendent, three were senior nursing officers, five were nursing officer or midwife level and one was a deputy director of nursing rank. Further details on healthcare workers interviewed are shown in Additional file 3.

A total of 66 clients age between 18 and 50 years (mean = 33.2 years) were involved in in-depth interviews of which 30 were men and 36 were women. Thirty-six were married, 23 were single, six were cohabiting with partners, one was single but in an intimate relationship and one was divorced. The majority of respondents (53) had completed high school and of Christian religion (58). All except three respondents reported occupations that generated income. All but three women reported at least one pregnancy. Further details on IDI study participants are shown in Additional file 4.

The 68 respondents for FGDs included community members age between 16 and 56 years with a mean of 32 years. Most of them had completed high school level education. All but 19 respondents (mostly adolescents) were occupied with income generating activities.

Other details on in-depth interview participant characteristics are show in Additional file 5.

### Knowledge of COVID-19, risk and prevention

Qualitative data showed that most participating clients had good understanding of aspects of COVID-19 such as the causes, mode of transmission, signs and symptoms. This knowledge was largely sourced from health promotion vans, interviews with healthcare personnel on radio, television and audios sent via social media. By endline, some misconceptions reported at baseline had given way to more extensive knowledge of the virus including its genetic make-up, and incubation period. All HCP interviewed were very knowledgeable about COVID-19.

### SRH service utilisation by clients

Survey data showed that the majority (68%) of clients visited the health centre for contraceptive/family planning services while none interviewed sought care for Gender Based Violence (GBV). Other details of client attendance are shown in Table 4 below.

The majority (71%) of clients seeking abortion care needed post-abortion care. More specifically, they needed care for incomplete abortion (81%) and or haemorrhage (78%). About 6% of PAC needed was for post abortion sepsis. The most popular method of abortion or treatment given was manual vacuum aspiration (52%), while dilation and curetage was the least (2%) frequent treatment given.

About 18% were screened for GBV and 41% of clients reported being screened for STIs during their visit. See Table 3 for further details.

**Table 3:** SRH Service utilisation by clients

**Characteristic**	Frequency	Percentage
Reason for visit (*N* = 990)>		
Abortion care (PAC & Induced)	52	5.2
Antenatal care	55	5.5
Delivery/postnatal care	3	0.3
Contraceptive/FP services	675	67.8
STI care	22	2.2
Other®	135	13.6
Type of abortion care sought during Visit (*n* = 52)		
Induced Abortion	16	28.8
Treatment Complications of Abortion	36	71.2
PAC needed/indicated (n = 36)		
Bleeding/Haemorrhage	28	77.8
Sepsis/Infection	2	5.6
Incomplete abortion	29	80.6
Counseling	1	4.8
Treatment/Method of abortion (n = 50)		
Manual Vacuum Aspiration	26	52.0
Electric Vacuum Aspiration	7	14.0
Dilatation And Curettage	1	2.0
Misoprostol	14	28.0
Mifepristone^b^	4	8.2
**Received contraceptive counselling during visit for abortion care**		
Yes, counselling only	27	52.9
Yes, counselling and method^a^	3	5.9
Was screened for gender-based violence (*n*** = **986)	183	18.6
Was screened/counselled for STI during visit (*n* = 255)	102	40.8

®Includes cervical cancer screening, other SRH care; ^a^Progestin injectable = 1, implant = 2; ^b^mifepristone is usually used in combination with misoprostol and not routinely used alone

Clients seeking various SRH services other than ANC and contraceptive services were assessed on their sexual risk behaviours. More than half (57%) of respondents had had oral sex with an intimate partner in the past three months and only about 14% used some form of protection. Also, 13.8% of respondents reported using protection the last time they had anal/vaginal sex.

Interviews with HCPs suggests that demand for abortion services was generally low with no change in the demand for abortion services during and after the pandemic. Provider perspectives suggest that abortion care was available and considered essential, yet, demand was low. They however explained that medical abortion was mostly prescribed for early pregnancies, especially during the lockdown period when non-emergency invasive procedures were fully suspended.

Qualitative interviews with clients and their partners revealed their need for contraception, especially for spacing, during the period and utilized existing services. These included counseling, continuation of existing methods and switching to other methods of contraception. The main reason given for switching methods was the experience of side effects. Also, female participants reported increased use of condoms and emergency contraceptive pills during the pandemic.

### Client utilisation of SRH services during the pandemic

**Fig. 1:**
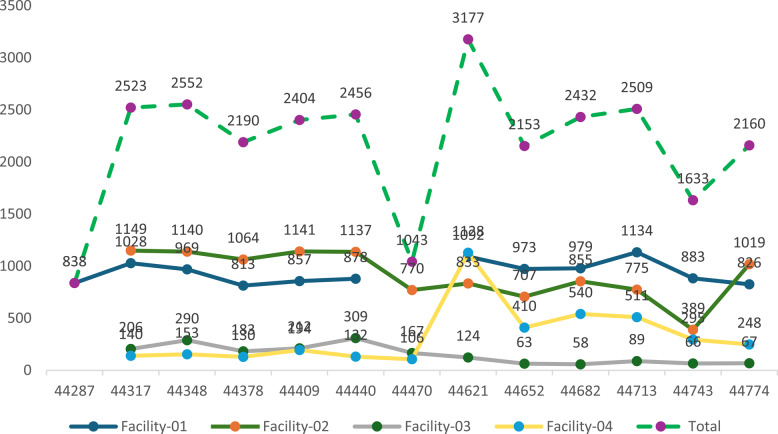
Number of clients visiting each health facility over observation period

Figure 1 below shows client attendance to selected facilities 6 months prior to baseline and endline visits. The baseline assessment shows similar attendance figures for starting and ending months (October and April 2021) and a decline in July 2021. Hence, attendance to all health facilities in May 2021 was higher than April 2021, whiles attendance in September 2021 was higher than October 2021.

Similarly, at end line, July 2022 recorded the least utilisation of SRH services in all health facilities within the 6-month period studied (March 2022 to August 2022). Health facilities 2, 3 and 4 recorded almost a half of client attendance compared to the same time (July) baseline period. There seemed to be rapid increase in client utilisation in facility 4 between October 2021 and March 2022 which is not observed in in other facilities.

### Service availability and readiness assessment in health facilities

An assessment of the readiness of the health facilities assessed showed that three of four were functioning as normal during baseline with the facility reporting disruption being one of the district hospitals or the polyclinic. The regional hospital did not report a disruption in any of their services both at baseline and enrolment. At endline, all health facilities reported normal functioning of outpatient, in-patient and pre-hospital emergency care services. Other details on facility readiness to provide services are shown in Table 4.

**Table 4:** Readiness of health facilities to provide services

	Baseline			Endline		
	Functioning as normal	Limited access	Don't know	Functioning as normal	Limited access	Don't know
Outpatient services	3/4	1/4	0/4	4	0/4	0/4
Inpatient services	3/4	1/4	0/4	4	0/4	0/4
Emergency unit services	3/4	1/4	0/4	3/4	1/4	0/4
Prehospital emergency care services (e.g. ambulance transport)	3/4	1/4	0/4	4/4	0/4	0/4
Community based care	3/4	1/4	0/4	2/4	2/4	0/4
Mobile clinics	2/4	1/4	1/4	3/4	0/4	1/4

Qualitative assessment of health service continuation over the period showed that that a full range of services were available during the pandemic period even though most facilities prioritised their resources first to the containment of COVID-19, especially during the lockdown. Below is a quotation from in-depth interview with healthcare workers:

*We did not stop any sexual reproductive health services. Rather, we strengthened the service and made the provision for women to be able to go through their normal delivery processes in terms of maternal health, where they had to do deliveries. We made a special room for mothers, who are found to be COVID-19 positive, where they would do their deliveries. We put measures in place, the theatre was constantly fumigated, you know. So, we did not stop maternal health or sexual reproductive health management because of the pandemic. We rather strengthened the health care delivery with respect to sexual reproductive health.* IDI 01_District Hospital.

Obviously, human resource was impacted with COVID-19 infections among healthcare workers leading to a shift system and or reduction in staff strength in all units of the hospital including SRH.

*We run shift. We were running shifts, yes. We reduced the nurses at the facility at that time. Others stayed home. So, you would stay home for one week. Your colleague comes to work for one week. Then the following week, you change over, yes. ….* IDI_District hospital_FP Unit.

*Well, a couple of my staff contracted the disease, so those staff members did not come to work. So, that is a reduction in the human resource capacity and strength. Also, there were a lot of, you know, fears about the disease that we had to do a lot of training and communication to alleviate the fears of my staff. But generally, work continued. It did not have any negative effects on their ability to work.* IDI 02 District Healthcare provider.

### Access to essential medicines and supplies

Qualitative data showed that there was no shortage of essential medicines and medical supplies in the facilities during the outbreak of COVID-19 as showed in the accounts below:

*It was always available, so there was no shortage, no complains, and even people had to come from other facilities because when they go, there is no one there. Our pharmacies were always stocked as well. We didn’t really have a problem with that.* IDI_Family planning.

*Generally, access to medicines are the same. As mentioned earlier, we rely on the regional medical stores for the essential medicines and procure unavailable ones from outside. Even though access can be made difficult by the exorbitant prices, we still manage to get all the essential medicines. *IDI 02 District Hospital.

However, there were reports of reduced financing for other necessary logistics that support healthcare delivery. These included oxygen and PPEs, and availability of these were heavily reliant on the size and availability of internally generated funds. During endline interviews, heads of facilities still reported financial distress Table 5.

**Table 5:** Effect of COVID-19 on General service provision and utilisation in health facilities

	Baseline	Endline
Actions/directives	Yes	Yes
Closure of outpatient services as per government directive	0/4	0/4
Closure of outpatient disease specific consultation clinics	1/4	0/4
Closure of population level cervical cancer screening programs	0/4	0/4
Decrease in outpatient volume due to patients not presenting	2/4	2/4
Decrease in inpatient volume due to cancellation of elective care	1/4	1/4
Inpatient services/hospital beds not available	3/4	0/4
Insufficient staff to provide services	2/4	1/4
Related clinical staff deployed to provide COVID‐1/49 relief	2/4	1/4
Insufficient Personal Protective Equipment (PPE) available for health care providers to provide services	2/4	2/4
Unavailability/Stock out of essential medicines, medical diagnostics or other health products at health facilities	1/4	1/4
Changes in treatment policies for care seeking behaviour for fever symptoms (e.g. stay at home policies)	2/4	1/4
Government or public transport lockdowns hindering access to the health facilities for patients	3/4	2/4
Financial difficulties during outbreak/lock down	3/4	2/4
Other (what are the other causes of this disruption and/or changes in service utilization):	1/4	2/4

*We still haven’t recovered. The impact of COVID has been so massive. We spent a lot of our resources purchasing the basic PPEs (Gloves and masks). At a point, the cost of purchasing items increased tremendously. For instance, the price for one of the PPEs moved from Gh₵ 8 to Gh₵ 350 per pack. We had limited resources and our income dwindled because of low attendance. We are currently still [dealing] with debts- that is why I mentioned earlier that we have not come out of it yet*. IDI 02 District Healthcare provider.

### Effect of COVID-19 on family planning services

Regarding facility readiness to provide FP services, three out of four facilities reported no disruption at all at both baseline and endline, while one facility reported a partial disruption for baseline and endline. Human resources for FP service provision ranged between two and seven at baseline with a median number of 3 providing services in the unit. At endline, there were increases in available staffing for the FP units ranging between three and twelve with a median of six staff manning the FP unit. Other details on FP service provision are shown in Table 6 below.

**Table 6:** Health facility availability and readiness to provide Family Planning services

	Baseline	Endline
	Yes	Yes
National family planning guidelines present in the facility?	2/4	4/4
any family planning check-lists and/or job-aids available in the facility?	4/4	4/4
**Infrastructure of the facility**		
Clear signs in the clinic on days and times in which services are available:	3/4	4/4
opening hours are convenient for clients, especially women and girls from key populations, including adolescents:	4/4	3/4
There is a reception desk at the facility to help inform and guide clients	3/4	4/4
There is a separate room for FP/contraception services	3/4	4/4
There are separate waiting rooms, especially for adolescents	2/4	0/4
The counselling rooms are curtained-off from others to listen and hear	3/4	4/4
The examination rooms are curtained-off from others to listen and hear:	4/4	4/4
There are simple seating and waiting areas for users sheltered from sun, wind and rain:	4/4	4/4
There are written information and materials available on the various contraceptive methods, so that users can take materials home to read	2/4	0/4
Clients contact information	4/4	4/4
Relevant medical history of the client	3/4	4/4
History of contraceptive method use	3/4	4/4
There are posters about violence against women	2/4	1/4

None of the health facilities had the full stock of reproductive health medicines and commodities at both baseline and endline. However, all facilities had a peculiar stock set which include combined estrogen progesterone oral contraceptive pill, progestin only contraceptive pill, progestin only injectable contraceptive (DMPA or NET-EN), cycle beads for standard days method, implant and copper IUDs. No facility had stock of female condoms, vaginal rings, levonorgestrel intrauterine device. Only 2 facilities had stock of male condoms at both baseline and endline. Male sterilization was available in only one facility at baseline but became more available in ¾ facilities at endline. However, female sterilisation was available in ¾ facilities at baseline but in all facilities at endline. Emergency contraceptive pill was available in ¼ facilities at baseline nut increased to 2/4 by endline.

Only one facility reported receipt of training for family planning service providers in the past 6 months prior to baseline and endline respectively.

### Effect of COVID-19 on abortion services

**Fig. 2:**
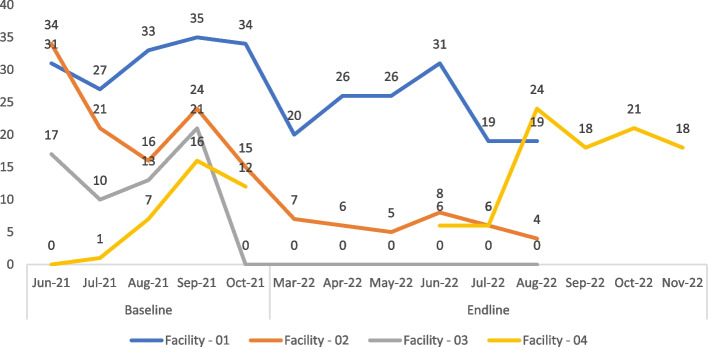
Client utilisation of abortion services over the study period (June 2021 – Nov 2022)

Similarly, safe abortion care services were not disrupted in three of the four facilities at both time points. One facility reported a partial disruption while the other could not report the status as its abortion services was integrated into general gynae care, which was not disrupted overall. Again, post-abortion care services were not disrupted in all facilities at baseline. However, one facility reported a partial disruption at endline. Client utilization of abortion services is shown in Fig. 2 below.

All facilities reported convenient opening hours for clients, and none of them reported referral of clients to other facilities for abortion services. Clear signs in the clinic regarding clinic days and times were available in 2/4 and ¾ facilities at baseline and endline respectively. At baseline ¾ facilities reported having a reception desk to inform and guide clients but at endline, all facilities had a functional reception desk. At baseline and endline, 2/4 and ¾ facilities respectively reported having separate rooms for abortion service clients. However, none of them had separate waiting rooms for adolescents. Regarding availability of commodities for abortion services, the three facilities that have abortion services distinct from general gynae department operations continued to stock up essentials over the pandemic period. Table 7 below reports on specific aspects of abortion service provision in the selected health facilities.

**Table 7:** Health facility availability and readiness to provide abortion services

	**Baseline**	**Endline**
	Yes	Yes
The counselling rooms are separate for private and confidential consultation:	2/4	4/4
The examination rooms are separate for private and confidential consultation:	3/4	4/4
There are simple seating and waiting areas (e.g. for users sheltered from sun, wind and rain):	4/4	4/4
There are written information and materials available on the various safe abortion methods, so that users can take materials home to read:	1/4	2/4
Clients contact information	4/4	3/4
Relevant medical history of the client	4/4	3/4
History of abortion service use:	4/4	3/4
Does the facility continue to stock abortion commodities?	3/4	3/4
22 gauge spinal needles for paracervical block:	2/4	3/4
21 gauge needles for drug administration:	3/4	3/4
Syringes 5 ml:	3/4	3/4
Syringes 10 ml:	4/4	3/4
Syringes 20 ml	4/4	2/4
IV (intravenous) line:	4/4	3/4
Blood pressure equipment:	4/4	4/4
Stethoscope	4/4	4/4
Speculum:	4/4	4/4
Tenaculum:	4/4	4/4
Tapered dilators up to 51 mm or equivalent circumference:	4/4	3/4
Electric vacuum aspirator (with 14 or 16 mm cannulae):	0/4	2/4
MVA aspirator and cannulae up to 12 mm:	4/4	4/4
Uterine evacuation forceps:	3/4	2/4
Large, postpartum flexible curette	3/4	3/4
Stainless steel bowl for preparing solution:	4/4	3/4
Instrument tray:	4/4	4/4
Clear glass dish for tissue inspection:	0/4	3/4
Oxygen and Ambu bag:	3/4	4/4
On-site access to an ultrasound machine (optional in some settings):	1/4	4/4
Long needle‐driver and suture:	4/4	2/4
Scissors:	4/4	4/4
Uterine packing:	4/4	3/4
Blood bank:	1/4	4/4
Misoprostol:	2/4	2/4
Osmotic dilators:	0/4	1/4
Mifepristone:	1/4	1/4
Analgesics:	2/4	2/4
Anxiolytics:	1/4	1/4
Fluids (saline, sodium lactate, glucose):	3/4	2/4
Appropriate antagonists to medications used for pain:	3/4	2/4
Lidocaine for paracervical block:	3/4	2/4
Uterotonics (oxytocin, misoprostol or ergometrine):	3/4	2/4
Antiseptic solution (non‐alcohol based) to prepare the cervix:	3/4	3/4
Sterilization or high‐level disinfection solutions and materials:	3/4	3/4
Clean examination gloves:	1/4	2/4
Gown:	1/4	3/4
Face protection	1/4	2/4
Clean water:	1/4	1/4
Detergent or soap:	1/4	2/4
Instrument soaking solution:	1/4	2/4
Gauze sponges or cotton balls:	1/4	2/4
Sanitary napkins or cotton wool:	0/4	2/4
Strainer (metal, glass or gauze):	1/4	2/4
Adequate toilet facilities:	1/4	2/4
Printed information for clients on post‐procedure self‐care:	3/4	2/4
Clear referral mechanisms to higher‐level facility, when needed:	0/4	3/4
Private area with chairs separate from antenatal or labour care, for women who wait in clinic for expulsion:	3/4	2/4
Have the abortion service providers received any training in safe abortion services in the last 6 months?	0/4	3/4
Have the abortion service providers received any training in adolescent sexual and reproductive health (including family planning) in the last 6 months?	0/4	3/4
Total number of human resources (staffing) in provision of safe abortion services in the past 2/4 months:	0/4	0/4
Gender of service providers available?	0/4	0/4
OB/Gyn specialist:	4	2/4
Medical doctor/general practitioner:	4	2/4
Midwife	2/4	3/4
Midwife:	3/4	4
Other health workers (recognized and trained by government e.g. Social worker, Counselor…):	3/4	2/4

### Barriers to SRH service utilization

Interviews with clients and their partners showed that there was widespread fear of COVID-19 infection due to the rapid spread recorded leading to the lockdown. For most clients, they took precautionary infection-control measures and accessed family planning and abortion services they considered urgent such as side effects. However, there were also substantial client reports of suspending health facility visits during the lockdown until the restriction on movement was lifted. Fear of COVID-19 infection remained the most frequently mentioned challenge to accessing SRH services during the pandemic. Nevertheless, most female respondents reported service utilization even in the face of the fear. At the endline interviews, fear of infection remained the key challenge mentioned by clients and their partners.

“*Yes, we those on the Depo, you have to be coming every three months, when your date is due, you are scared to come, but you need to. So, when you come here and you get to the main entrance, without a nose mask, you will not be allowed to enter, and you also have to wash your hands*”. IDI 07, client.

Other barriers to utilisation mentioned include restriction on movements, especially with military presence, and other social reasons such as relocation to new areas.

### Mitigating factors

Our study found two major innovations by healthcare managers to sustain SRH care provision during the COVID-19 pandemic. These included the low-cost production of PPEs and the introduction or expansion of Telemedicine.

Small-scale production of PPEs in health facility was mobilized by health staff using internally generated funds to address issues of PPE shortages. Hand sanitizers and disinfectants were produced by the hospitals’ pharmacy departments while layered cloth face masks were produced with the support of volunteers. Some of these face masks were sold to the public to raise funds for the health facilities.

Three out of four health facilities had telemedicine facilities already in place during baseline. The district hospital that did not have the facility established it by endline. All facilities had to use task shifting to make up for the staff either posted to COVID-19 treatment and detention facilities or for more clinical roles. Other actions taken to maintain health services are shown in Table 8.

**Table 8:** Mitigating actions taken by health facilities

	Baseline	Endline
	Yes	Yes
Telemedicine deployment to replace in‐person consultations	3/4	4/4
Task shifting/role delegation	4/4	3/4
Novel supply chain and/or dispensing approaches for medicines through other channels	3/4	3/4
Triaging to identify priorities	3/4	4/4
Redirection of patients to alternate health care facilities	2/4	1/4
Community outreach to inform on service disruptions and changes	4/4	2/4
Government removal of user fees	1/4	0/4
Other (describe what other approaches are being used)	0/4	0/4

## Discussion

The disruption in FP and abortion services observed in this study is consistent with evidence from across the globe [13]. The fluctuating patterns of utilization observed in this current study were very much aligned with the waves of COVID-19 experienced in Ghana. Even though the qualitative evidence suggested that the lockdown period was the most impactful due to fear of infection and the restriction on physical movements, the effect of the first major wave in June-July 2020 was acknowledged by both healthcare workers and clients. Unfortunately, none of our qualitative data collection time points coincided with any of the four subsequent waves and therefore cannot explain the lower utilizations around the peaks of infection. Nevertheless, qualitative evidence at endline suggested that the general population anxiety and fear of infection was greatly reduced with the later peaks of infection recorded in Ghana. Due to the special and prefecture level differences in participating facilities, varying levels of effect was observed. For instance, data showed that the disruptions were more evident in one of the district hospitals and the polyclinic rather than the regional hospital and the relatively more resourced district hospital. Considering that fear of COVID-19 infection was cited as a major reason for non-attendance, the regional hospital which doubled as the lead national COVID-19 treatment center for the country ironically did not report a disruption of services. It is possible that client non-attendance may have been influenced by their perceived lack of availability of staff for non-emergency services, which was particularly more pronounced among clients recruited from facilities that reported service disruptions due to low staff strength. Another possible reason for low utilization with the restoration of services post-pandemic period is the inadequacy of information that meets client needs. This is especially worth investigating since three health of the four facilities indicated the availability of clear signs that services were available.

This study found that abortions were generally low compared to family planning and this is consistent with existing evidence in Ghana. Both health service providers and clients accounts indicated health facilities’ readiness and availability to provide abortion services during the lockdown and the period after even with the temporary suspension of non-emergency surgical procedures including surgical abortion. Our finding that healthcare providers were ready to provide surgical interventions for post abortion care but not for initiations is backed by existing evidence rooted in stigmatization [14]. They were more poised to provide medical abortion services under the circumstances. Stigma remains a key barrier to seeking and provision of abortion care in most settings including Ghana [14, 15]. Both client and provider interviews revealed that stigma had a negative impact on women and couple’s decision to have an abortion. This calls for widespread in-service training of healthcare staff, especially on values clarification and attitudinal transformation(VCAT) to eliminate this major barrier [16]. Aside from the stigma and fear of COVID-19 infection, the low utilization of abortion services could be that clients needing it rather procured medical abortion pills from pharmacies and chemical shops which are a well-documented source of the pills [17–20]. There is opportunity to use telemedicine for abortion services to eliminate some barriers for women [21]. Evidence from Scotland on women’s view of telemedicine abortion services showed very high acceptability and promised sustainability of the programme beyond COVID-19 [22]. Also, clients may have turned to private health facilities during the period due to the perception of relatively fewer patients and staff readiness to provide service compared to the public facilities that have to divide attention to COVID-19 treatment. Nevertheless, qualitative accounts showed that women knew that they could always seek post abortion care at the health facilities if there were complications requiring invasive methods such as incomplete abortion or haemorrhage.

Resilience remains a critical expectation of health systems across the globe to control the pandemic and also maintain delivery of existing services [23]. Financial stress was reported by healthcare providers during the pandemic period even though the Government of Ghana had allocated funds for the fighting of COVID-19. The resultant shifts of resources to fighting COVID-19 led to increased pressure on managers to sustain full running of their facilities with attenuated internally generated funds resulting from reduced client attendance. Our study found innovations such as self-production of PPEs at a relatively lower cost to support budgetary needs. Another innovation implemented by healthcare managers was the improvement in telemedicine facilities and its institution in a health facility that did not have one prior to COVID-19. While this was a show of great commitment of the facilities to reach their clientele, the services were general and not focused on SRH. Meanwhile, known clients were routinely followed up with telephone calls by the providers at the family planning clinic prior to and during the pandemic. It would be valuable to include SRH in the telemedicine service provision or at least expand the telephone consultations beyond their existing clientele. While efforts are being made to improve telemedicine facilities across health facilities, instituting a call center to attend to SRH needs such as information, counseling and non clinic based care for family planning and abortion would potentially yield benefits as in the Malian context [24].

This study has showed that there was some disruption of family planning and abortions services during the COVID-19 pandemic in Ghana. While the disruption was short and relatively less impactful due to the relatively low infection rates and very short lock-down period, the sensitivity of client utilization to the waves of infection in the country suggest that the picture would be been dire with prolonged and high infections. Also, the inclusion of SRH in the list of essential services may have been beneficial in the commitment to sustain SRH services throughout the pandemic. Furthermore, qualitative evidence showed that there was no disruption in maternity services in all facilities, made possible by the wide circulation of guidelines for maintaining services at all levels of care. While this study did not assess client utilization of maternity services to know the impact of the guidelines, the interviews showed that maternity services were not interrupted because there were specific national guidelines on service provision during the covid. This was not the case for all SRH services during pandemic situations.

### Strengths and limitations

This study has some strengths worth discussing. First, focusing the study in the Greater Accra region of Ghana [home to the nation’s capital], which recorded the highest number of COVID-19 infections and deaths in the country this study, also happens to be the region most resourced with specialised healthcare staff in the country. This affords Ghana the opportunity to ascertain its capacity to contain pandemic situations in the best-case scenario in terms of human resource, logistics and health administration compared to other parts of the country. Second, the use of both quantitative and qualitative approaches to studying the health system’s readiness, availability and utilisation of SRH services gives a more holistic view of the situation. Third, this study involved different levels of health facilities serving rural, urban and peri-urban population, and all socioeconomic groups, allowing a more holistic understanding of health system response and client behaviour during pandemic situations.

Another limitation of the current study is its exclusion of some possible sources of contraception and abortion service provision in the country, such as private health facilities, pharmacies and chemical outlets. It was therefore impossible to report on the extent to which these sources were used during the pandemic. Also, the findings of this study may not be generalizable to other parts of the country with different population characteristics and health system settings.

## Conclusions

This study concludes that health facilities were generally ready to provide SRH services during the COVID-19 period, yet, some clients could not access the services for varying reasons. All SRH services were available in health facilities except that a temporary suspension of surgical abortion methods was reported during the lockdown period. However, abortion related complications were treated promptly even if they required surgical methods. FP service utilisation was high compared to abortion services. Telemedicine services available at the facilities offered general healthcare services and not tailored to meet SRH care needs.

Knowledge on COVID-19 was high among both clients and service providers and were aware of the risk of COVID-19 infection while accessing care at the health facility. Qualitative data showed that both clients and providers perceived that quality services were available during the pandemic. The fear of infection and stigma influenced the seeking for abortion care.

Client attendance to FP and abortion services as well as reported availability of commodities showed a slight disruption in service provision, but this was very short-lived. A comparison of baseline and endline data showed that health facilities had fully recovered from the initial shocks of the pandemic.

The current low impact of the COVID-19 on SRH service provision is explained by the relatively lower infection recorded in the country compared to other country settings. However, national COVID-19 preparedness strategy divulged well ahead of the first recorded case in Ghana can be credited for the attenuated impact of COVID-19 observed. Consequently, the team work at various levels of the health system was a key driver of the success recorded.

## Disclaimer

This report contains the collective views of an international group of experts and does not necessarily represent the decisions or the stated policy of WHO.

## Supplementary Information

Supplementary Material 1

Supplementary Material 2

Supplementary Material 3

Supplementary Material 4

Supplementary Material 5

## Data Availability

The data will be available upon request as per the WHO policies. Requests for access to data can be sent to alimoa@who.int.

## Abbreviations

FP: Family Planning
SARA: Service Availability and Readiness Assessment
COVID-19: CoronaVIrus Disease of 2019
HCP: Healthcare Providers
SRH: Sexual and Reproductive health

## References

[1] Nana-Sinkam P, Kraschnewski J, Sacco R, Chavez J, Fouad M, Gal T, et al. Health disparities and equity in the era of COVID-19. J Clin Transl Sci. 2021. 10.1017/cts.2021.23. 34192054 10.1017/cts.2021.23 PMC8167251

[2] Winkelmann J, Webb E, Williams GA, Hernández-Quevedo C, Maier CB, Panteli D. European countries’ responses in ensuring sufficient physical infrastructure and workforce capacity during the first COVID-19 wave. Health Policy. 2022. 10.1016/j.healthpol.2021.06.015. 34311982 10.1016/j.healthpol.2021.06.015 PMC9187509

[3] Larkin HD. COVID-19 limited access to sexual and reproductive health services. JAMA. 2022. 10.1001/jama.2022.18476. 36378199 10.1001/jama.2022.18476

[4] UNFPA. Accelerating the promise: the report on the Nairobi Summit on ICPD25. New York: UNFPA; 2020.

[5] World Health Organisation. Sexual and reproductive health and rights in the context of COVID-19 in the African Region: Rapid assessment of continuity of services. 2021.

[6] UNFPA. The COVID Chronicles, UNFPA’s COVID-19 Response in Ghana [Available from: https://ghana.unfpa.org/en/publications/covid-chronicles-unfpas-covid-19-response-ghana. 2020.

[7] Banke-Thomas A, Yaya S. Looking ahead in the COVID-19 pandemic: emerging lessons learned for sexual and reproductive health services in low- and middle-income countries. Reprod Health. 2021. 10.1186/s12978-021-01307-4. 34906177 10.1186/s12978-021-01307-4 PMC8670615

[8] Agbozo F, Jahn A. COVID-19 in Ghana: challenges and countermeasures for maternal health service delivery in public health facilities. Reprod Health. 2021. 10.1186/s12978-021-01198-5. 34281582 10.1186/s12978-021-01198-5 PMC8287110

[9] Biney AAE, Kayi E, Atiglo DY, Sowah LR, Badasu D, Ankomah A. COVID-19, relationships, and contraception: Qualitative perspectives from emerging adults during the COVID-19 lockdown in Accra, Ghana. SSM - Qualitative Research in Health. 2023;3. 10.1016/j.ssmqr.2022.100216 PMC9789569 36589527

[10] Fuseini K, Jarvis L, Hindin MJ, Issah K, Ankomah A. Impact of COVID-19 on the Use of Emergency Contraceptives in Ghana: An Interrupted Time Series Analysis. Frontiers in Reproductive Health. 2022;4. 10.3389/frph.2022.811429 PMC9580762 36303651

[11] Ghana Health Service. District health information management system II (DHIMSII): Family planning acceptor rates. 2024.

[12] Kouanda S, Nahyuha Chomi E, Kim C, Jen S, Bahamondes L, Cecatti JG, et al. Health systems analysis and evaluation of the barriers to availability, utilisation and readiness of sexual and reproductive health services in COVID-19-affected areas: a WHO mixed-methods study protocol. BMJ Open. 2022. 10.1136/bmjopen-2021-057810. 35649598 10.1136/bmjopen-2021-057810 PMC9160592

[13] Vanbenschoten H, Kuganantham H, Larsson EC, Endler M, Thorson A, Gemzell-Danielsson K, et al. Impact of the COVID-19 pandemic on access to and utilisation of services for sexual and reproductive health: a scoping review. BMJ Glob Health. 2022. 10.1136/bmjgh-2022-009594. 36202429 10.1136/bmjgh-2022-009594 PMC9539651

[14] Aniteye P, O'Brien B, Mayhew SH. Stigmatized by association: challenges for abortion service providers in Ghana. BMC Health Serv Res. 2016;16(1):486. 10.1186/s12913-016-1733-7. 10.1186/s12913-016-1733-7 PMC5018197 27612453

[15] Payne CM, Debbink MP, Steele EA, Buck CT, Martin LA, Hassinger JA, et al. Why women are dying from unsafe abortion: narratives of Ghanaian abortion providers. Afr J Reprod Health. 2013;17. 24069757

[16] Turner KL, Pearson E, George A, Andersen KL. Values clarification workshops to improve abortion knowledge, attitudes and intentions: A pre-post assessment in 12 countries. Reprod Health. 2018;15. 10.1186/s12978-018-0480-0 PMC5838872 29506542

[17] Ganle JK, Busia NT, Baatiema L. Stocking and over-the-counter sale of misoprostol for medical abortion in Ghana’s community pharmacies: comparison of questionnaire and mystery client survey. Int J Pharm Pract. 2020. 10.1111/ijpp.12593. 31746501 10.1111/ijpp.12593

[18] Ganle JK, Busia NT, Maya E. Availability and prescription of misoprostol for medical abortion in community pharmacies and associated factors in Accra, Ghana. Int J Gynaecol Obstet. 2019. 10.1002/ijgo.12717. 30451283 10.1002/ijgo.12717

[19] Otsin MNA, Black K, Hooker L, Taft AJ. Pharmacy dispensing of abortion pills in Ghana: experiences of pharmacy workers and users. BMJ Sex Reprod Health. 2023;49(4):254–9. 10.1136/bmjsrh-2022-201674. 10.1136/bmjsrh-2022-201674 36944481

[20] Agula C, Henry EG, Asuming PO, Agyei-Asabere C, Kushitor M, Canning D, et al. Methods women use for induced abortion and sources of services: insights from poor urban settlements of Accra, Ghana. BMC Womens Health. 2021. 10.1186/s12905-021-01444-9. 34399739 10.1186/s12905-021-01444-9 PMC8365972

[21] Oyediran KA, Makinde OA, Adelakin O. The role of telemedicine in addressing access to sexual and reproductive health services in sub-saharan africa during the covid-19 pandemic. Afr J Reprod Health. 2020;24 2 Special Edition COVID-19. 10.29063/ajrh2020/v24i2s.8 34077053

[22] Boydell N, Reynolds-Wright JJ, Cameron ST, Harden J. Women’s experiences of a telemedicine abortion service (up to 12 weeks) implemented during the coronavirus (COVID-19) pandemic: a qualitative evaluation. BJOG. 2021. 10.1111/1471-0528.16813. 34138505 10.1111/1471-0528.16813 PMC8441904

[23] Pradhan NA, Samnani AABA, Abbas K, Rizvi N. Resilience of primary healthcare system across low- and middle-income countries during COVID-19 pandemic: a scoping review. Health Res Policy Syst. 2023;21(1):98. 10.1186/s12961-023-01031-4. 10.1186/s12961-023-01031-4 PMC10507852 37723533

[24] Haidara FC, Keita AM, Ducker C, Diarra K, Djiteye M, Marlow H, Goodwin E, Martell O, Izugbara C, Sow S. Provision and uptake of sexual and reproductive health services during the COVID-19 pandemic: the case of Mali. Afr J Reprod Health. 2022;26(12s):169-79. 10.29063/ajrh2022/v26i12s.18. 10.29063/ajrh2022/v26i12s.18 37585172

