# Supplementary material for: Family planning and abortion service availability and utilisation during the COVID-19 pandemic in Ghana
Source: Reprod Health. 2025 Nov 20;22(Suppl 3):234. doi: 10.1186/s12978-025-02122-x (PMC12632033; doi:10.1186/s12978-025-02122-x)
Supplement: Supplementary file 1 — Supplementary Material 1 [file 12978_2025_2122_MOESM1_ESM.pdf]

Project ID: A 6 6 0 0 7 Subject number: ____

Country: ____________ Center ID: ____

GENERAL

1. Date questionnaire completed: Day __ Month __ Year ____

2. a) Date of birth: Day __ Month __ Year ____

or

2. b) Age: **years** __

3. Sex: ☐
   1 = Female 2 = Male 3. Other

4. a) Education Level: ☐
   0 = None
   1 = Primary (1-6)/Elementary 1 (1 - 5 years)
   2 = Secondary school/Elementary 2 (6 - 9 years)
   3 = High school-Vocational school/Secondary
   4 = University/college
   5 = Graduate school

   4. b) Number of school years achieved: **years** __

5. Marital Status: ☐
   1 = Single
   2 = Marital/Cohabiting
   3 = Separated
   4 = Divorced
   5 = Widowed

6. Identify your Ethnic group: __
   1 = Han
   2 = Caucasian
   3 = African
   4 = South Asian
   5 = East Asian
   6 = Latino/Hispanic
   7 = Middle Eastern
   8 = Other
   If ***Other*** , Specify: ________________________________

7. Are you currently smoking tobacco and/or living with someone who smokes? ☐
   1 = No
   2 = Yes, living with somene who smokes
   3 = Yes, smoking
   4 = Smoking and living with someone who smokes

8. Have you been tested for COVID-19 since December 2019? ☐
   1 = No (***Goto Q9***)
   2 = Yes
   3 = Refused to answer (***Goto Q9***)

   8.a) When was most recent test done? Day __ Month __ Year ____

   8. b) What type of test and what was the result on the COVID-19 test? *(Most recent)*
   0 = Not done 1 = Negative 2 = Positive
   1. PCR: ☐
   2. IgG/IgM: (Seological) ☐
   3. Antigenic: ☐

Project ID: A 6 6 0 0 7 — Subject number: [ ][ ][ ][ ]

Country: [ ][ ][ ][ ][ ][ ][ ][ ][ ][ ][ ][ ] — Center ID: [ ][ ][ ][ ]

PREGNANCY

If Q3= 'Male' go directly to Q11

9. Are you currently pregnant? [ ]
   1 = No   2 = Yes

10. How many pregnancies did you have excluding current pregnancy:

    10.a) Pregnancies: [ ][ ]

    10.b) Number of abortions: [ ][ ]

    10.c) Number of live births: [ ][ ]

    10.d) Number of living children: [ ][ ]

    10.e) Vaginal delivery: [ ][ ]

    10.f) C-section: [ ][ ]

11. What is the main reason for the current visit? [ ]
    1 = Post-abortion care and abortion care *(**Goto Q12 to Q17, and Q32**)*
    2 = Pregnancy / Antenatal care (***Goto Q12, Q16 to Q32*** *)*
    3 = Pregnancy / Delivery care *(**Goto Q12, Q16, Q 18 and Q32**)*
    4 = Postnatal care *(**Goto Q16, Q18 and Q32**)*
    5 = Contraception/family planning *(**Goto Q16, Q18 and Q32**)*
    6 = Help with violence at home *(**Goto Q16, Q18 and Q32**)*
    7 = Screening/treatment for STIs *(**Goto Q16 to Q32**)*
    8 = Abortion **(Goto Q12 to Q17, and Q32)**
    9 = Other *(* ***Specify, Q16 to Q32)***
    If ***Other***, specify: ______________________________

12. Gestational Age: [ ]
    1 = First trimester *(= **< 13 weeks**)*
    2 = Second trimester *(**between 14 and 27 weeks**)*
    3 = Third trimester *(**>= 28 weeks**)*

Project ID: | A | 6 | 6 | 0 | 0 | 7 |

Subject number: ____

Country: ____

Center ID: ____

ABORTION

13. a) Type of care/service for current abortion care visit:

1 = Induced abortion **(Skip *Q13c*)**

2 = Treatment of situations or complications of abortion (***Goto Q13b and c***)

3 = Other (Goto Q14)

If ***Other***, specify: ____

13. b) What treatment was offered?

1 = No 2 = Yes

1 . Manual vacuum aspiration:

2. Electric vacuum aspiration:

3. Dilatation and curettage:

4. Misoprostol:

5. Mifeprostone:

6. Other:

If ***Other***, specify: ____

13. c) *If treatment of complications of abortion, specify complication:*

1 = No 2 = Yes

1. Bleeding/Haemorage :

2. Uterine perforation:

3. Genital trauma:

4. Sepsis/infection:

5. Incomplete abortion:

5. Other:

If ***Other***, specify: ____

Project ID: A 6 6 0 0 7 Subject number: ☐☐☐☐

Country: ☐☐☐☐☐☐☐☐☐☐☐☐ Center ID: ☐☐☐☐

ABORTION (CONTINUED)

14. Additional post-abortion interventions provided:

1 = No (***Goto Q15***) 2 = Yes

14. a) Antibiotic treatment: ☐

14. b) Surgical management (including exploratory laparotomy, hysterectomy): ☐

14. c) Intensive care unit (continuous monitoring /medical supervision): ☐

14. d) Blood transfusion: ☐

14. e) Other: ☐

If ***Other*** , specify: ____________________

15. a) Did the woman receive any contraception care before hospital discharge? ☐

1 = No (***Goto Q16 and Q32***)

2 = Yes, family planning/contraception counselling only **(Goto Q16 and Q32)**

3 = Yes, counselling and contraceptive provision/ prescription/method application (***Goto Q15b and Q16 and Q32***)

15. b) If ***Yes counselling and contraceptive provision/ prescription/method application, which method?***

More than one answer possible ☐☐ ☐☐

1 = Combined estrogen progesterone oral contraceptive pill

2 = Progestin-only contraceptive pill

3 = Combined estrogen progesterone injectable contraceptive (CIC)

4 = Progestin-only injectable contraceptive (DMPA or NET-EN)

5 = Male condom

6 = Female condom

7 = Emergency contraceptive pill

8 = Cycle beads for standard days method

9 = Vaginal ring

10 = Male sterilization

11 = Female sterilization

12 = Implant (sub dermal implant)

13 = Copper IUD

14 = Levonorgestrel Intrauterine Device

15 = Other natural family planning method

16 = Lactational Amenorrhea Method

17 = Diaphragm

15. c) What type of contraception generally used by client, and reason for discontinuation? ____________________

16. Did anyone ask you about your situation at home, problems with your partner or if you have experienced any form of violence or abuse? ☐

1 = No (***Goto Q17***) 2 = Yes ***(Go to Q16a)***

16a. Did you receive any counseling or referral to counseling for problems at home? ☐

1 = No 2 = Yes

Project ID: A 6 6 0 0 7 | Subject number: ☐☐☐☐

Country: ☐☐☐☐☐☐☐☐☐☐☐☐ | Center ID: ☐☐☐☐

ABORTION (CONTINUED)

17. Did you receive any screening and/or counseling on STIs? ☐

1 = No   2 = Yes

CONTRACEPTION / FAMILY PLANNING

18. a) Did the client receive family planning/contraception services (including method) during this current visit? ☐

1 = No (***Goto to 19***)

2 = Yes, family planning/contraception counseling only (***Goto 18 b***)

3 = Yes, counseling and contraceptive method (***Goto Q18b***)

18. b) If **Yes**, which contraceptive method? ☐☐ ☐☐

More than one answer possible

1 = Combined estrogen progesterone oral contraceptive pill

2 = Progestin-only contraceptive pill

3 = Combined estrogen progesterone injectable contraceptive (CIC)

4 = Progestin-only injectable contraceptive (DMPA or NET-EN)

5 = Male condom

6 = Female condom

7 = Emergency contraceptive pill

8 = Cycle beads for standard days method

9 = Vaginal ring

10 = Male sterilization

11 = Female sterilization

12 =Implant (sub dermal implant)

13 = Copper IUD

14 = Levonorgestrel Intrauterine Device

15 = Other natural family planning method

16 = Lactational Amenorrhea Method

17 = Diaphragm

Project ID:	A 6 6 0 0 7	Subject number:	
Country:		Center ID:	

STI

19. In the past three months, have you had oral, vaginal, or anal sex with any person or people of the following genders? *[check all that apply]*

1 = No *2 = Yes*

Female **if "Yes"go to Q19a**

Male **if "Yes"go to Q19b**

I have had oral sex with any partners in the past three months

19. a) If you answered you had female partners: During the past three months, how many female sexual partners have you had? When we say sexual partners, we are referring to individuals with whom you have had oral, vaginal, or anal sex.

Female sexual partners

19. b) If you answered you had male partners: During the past three months, how many male sexual partners have you had? When we say sexual partners, we are referring to individuals with whom you have had oral, vaginal, or anal sex.

Male sexual partners

20. If you reported having a sexual partner of any gender in the past three months: Last time you had oral sex, did you use a condom or other form of protection?

1 = No *2 = Yes* *3 = Don't know*

21. If you reported having a sexual partner of any gender in the past three months: Last time that you had vaginal or anal sex, did you use a condom?

1 = No *2 = Yes* *3 = Don't know*

22. Have you ever been tested by a doctor or any other provider for a sexually transmitted infection (STI), other than HIV? STIs include things such as chlamydia, gonorrhea, and syphilis.

1 = No. **Go to Q26** *2 = Yes* **if "Yes"go to Q23 & Q24** *3 = Don't know.* **Go to Q26**

23. When was your most recent STI screening (not including HIV testing)?

1= Within the past 3 months

2= 3-6 months ago

3= 6-12 months ago

4= 1-2 year ago

5= 2-5 years ago

6= Over 5 year ago

24. In your lifetime, have you ever been told by a doctor or other provider that you had a sexually transmitted infection (STI), other than HIV?

1 = No **if "No", skip to Q26** *2 = Yes* **if "Yes", go to Q25** *3 = Don't know* **if "I don't know", skip to Q26**

Project ID: A 6 6 0 0 7 Subject number:

Country: Center ID:

STI (Continued)

25. With which sexually transmitted infection(s) were you diagnosed? [check all that apply]

1 = No 2 = Yes

25a) Anal warts (HPV)
25b) Chlamydia
25c) Genital warts (HPV)
25d)Gonorrhea
25e) Genital herpes
25f) Syphilis
25g) Trichomoniasis
25h) Mycoplasma genitalium
25i) Do not know the name of the infection

26. In the past three months, have you experienced any of the following symptoms? *[check all that apply]*

1 = No 2 = Yes

26a) Discharge from penis or vagina
26b) Pain during urination
26c) Sores/blisters in genital area
26d) Warts in the genital area
26e) Rash or itching in genital area

27. Thinking of your life at this moment, what do you think are your most important sexual health needs or concerns? *[Please select up to* ***three*** *options from the list below]*

1 = No 2 = Yes

27a) Access to condoms
27b) Pre-exposure prophylaxis (PrEP)
27c) HIV testing
27d) HIV treatment
27e) STI testing
27f) STI treatment
27g) Access to preferred method of contraception
27h) Abortion care
27i) Communication with sexual partner[s] about condoms
27j) Communication with my sexual partner[s] about HIV
27k) Communication with my sexual partner[s] about STIs
27l) Communication with my sexual partner[s] about contraception/pregnancy
27m) Communication with my healthcare provider[s] about sexual and reproductive health
27n) Sexual function (ex: erectile dysfunction, impotency, lack of arousal)
27o) Sexual satisfaction (how much you enjoy sex)
27p) Emotional intimacy
27q) Sexual coercion (partner or partners persuading you to have sex when you do not want to)
27r) Other

Project ID: A 6 6 0 0 7

Subject number:

Country:

Center ID:

STI (Continued)

28. Have you ever used this STIs service?

1 = Yes, in the last 12 months **go to Q29**

2 = Yes, more than a year ago **go to Q29**

3 = No **go to Q31**

4 = I don't remember **go to Q31**

29. Thinking of your last previous access (EXCLUDING this one), how difficult or easy was:

1 = Very difficult

2 = Difficult

3 = Moderate

4 = Easy

5 = Very easy

6 = Not Applicable

29. a) Getting an appointment

29. b) Seeing the doctor or nurse for a consultation

29. c) Getting lab tests for STIs

29. d) Getting the treatment for STIs

30. Did you have access to the STIs service during the COVID19-related lockdown period?

1 = No *2 = Yes* *3 = Don't know*

31. Considering the CURRENT STIs service you are accessing, how difficult or easy was:

1 = Very difficult

2 = Difficult

3 = Moderate

4 = Easy

5 = Very easy

6 = Not Applicable

31 a) Getting an appointment

31 b) Seeing the doctor or nurse for a consultation

31 c) Getting lab tests for STIs

31 d) Getting the treatment for STIs

COMMENT

32. Comment: ______________________

Interviewer's Initials: ______________ Interviewer's signature: ______________

Date General form completed:

Day		Month			Year			

First data entry (initials): ______________ Second data entry (initials): ______________

	A66007 - Health systems analysis and evaluations of the barriers to availability, utilization and readiness of sexual and reproductive health services in COVID-19 affected areas	HSC
World Health Organization	Health service continuation module	Page 1/4<br>V 0.20 (12 May 2021)

Project ID: A 6 6 0 0 7 Country: ☐☐☐☐☐☐☐☐☐☐☐☐

Center ID: ☐☐☐☐ Year of reported data: ☐☐☐☐ Month of reported data: ☐☐

GENERAL

1. Date of questionnaire:

Day		Month		Year			

POLICIES AND PLANS

2. Has your country defined a national SRH essential health services package (prior to the COVID-19 pandemic)? ☐
   1 = No 2 = Yes 3 = Don't know

3. Has your country identified a core set of essential health services to be maintained during the COVID-19 pandemic? ☐
   1 = No 2 = Yes 3 = Don't know

4. Is there additional government funding allocated to assuring essential health services? ☐
   1 = No 2 = Yes 3 = Don't know

5. Dissemination and use of WHO and other Technical Guidance documents related to COVID-19 response and continuity of SRH essential health services:

   5. a) Have you received latest guidelines? ☐
   1 = No ***(Goto Q6)*** 2 = Yes (Goto Q5 b) 3 = Don't know **(Goto Q6)**

   5. b) If *Yes* , have you shared latest guidelines with health service providers? ☐
   1 = No 2 = Yes

   5. c) Are the guidelines being used to guide the routine service provision? ☐
   1 = No 2 = Yes

MAINTENANCE OF ESSENTIAL HEALTH SERVICES

6. During the COVID-19 pandemic, what are the government policies for the following?
   1 = Functioning as normal 3 = Suspended
   2 = Limited access (e.g. limited service hours, or who can receive services) 4 = Don't know

   6. a) Outpatient services: ☐

   6. b) Inpatient services: ☐

   6. c) Emergency unit services: ☐

   6. d) Prehospital emergency care services (e.g. ambulance transport): ☐

   6. e) Community based care: ☐

   6. f) Mobile clinics: ☐

Project ID: A 6 6 0 0 7 Country: ☐☐☐☐☐☐☐☐☐☐☐☐

Center ID: ☐☐☐☐ Year of reported data: ☐☐☐☐ Month of reported data: ☐☐

MAINTENANCE OF ESSENTIAL HEALTH SERVICES (CONTINUED)

7. Which of the following services have been disrupted due to COVID-19?

1 = Completely disrupted	4 = Not applicable
2 = Partially disrupted	5 = Don't know
3 = Not disrupted	

7. a) Family Planning and contraception: ☐

7. b) Antenatal care: ☐

7. c) Safe abortion services: ☐

7. d) Post-abortion care: ☐

7. e) Facility based births: ☐

7. f) Gender based violence: ☐

7. g) Routine immunization services in health facilities: ☐

7. h) Sick child services/IMNCI: ☐

7. i) Outbreak detection and control (for non-COVID diseases): ☐

7. j) 24-hour emergency room/unit services: ☐

7. k) Urgent blood transfusion services: ☐

7. l) Inpatient critical care services: ☐

7. m) Emergency surgery (including obstetric, infection): ☐

7. n) Other (what other related services has been disrupted due to COVID-19): ☐

If ***Other*** , specify: ________________________________

Project ID: A 6 6 0 0 7 Country:

Center ID: Year of reported data: Month of reported data:

MAINTENANCE OF ESSENTIAL HEALTH SERVICES (CONTINUED)

8. What are the main causes of this disruption(s) and/or change(s) in service utilization?

1 = No 2 = Yes 3= Don't know

8. a) Closure of outpatient services as per government directive: ☐

8. b) Closure of outpatient disease specific consultation clinics: ☐

8. c) Closure of population level cervical cancer screening programs: ☐

8. d) Decrease in outpatient volume due to patients not presenting: ☐

8. e) Decrease in inpatient volume due to cancellation of elective care: ☐

8. f) Inpatient services/hospital beds not available ☐

8. g) Insufficient staff to provide services: ☐

8. h) Related clinical staff deployed to provide COVID-19 relief: ☐

8. i) Insufficient Personal Protective Equipment (PPE) available for health care providers to provide services: ☐

8. j) Unavailability/Stock out of essential medicines, medical diagnostics or other health products at health facilities: ☐

8. k) Changes in treatment policies for care seeking behaviour for fever symptoms (e.g. stay at home policies): ☐

8. l) Government or public transport lockdowns hindering access to the health facilities for patients: ☐

8. m) Financial difficulties during outbreak/lock down: ☐

8. n) Other (what are the other causes of this disruption and/or changes in service utilization): ☐

If ***Other*** , specify: ____________________

Project ID: A 6 6 0 0 7

Country:

Center ID:

Year of reported data:

Month of reported data:

9. What approaches are being used to overcome the disruptions to essential health services in public sector health facilities?

1 = No 2 = Yes 3 = Don't know

9. a) Telemedicine deployment to replace in-person consults (e.g. M Health, Hotlines, WhatsApp platforms, telephone consultations...): ☐

9. b) Task shifting / role delegation: ☐

9. c) Novel supply chain and/or dispensing approaches for medicines through other channels: ☐

9. d) Triaging to identify priorities: ☐

9. e) Redirection of patients to alternate health care facilities: ☐

9. e1) If yes, which alternate health care facilities: ______________

9. f) Community outreach to inform on service disruptions and changes: ☐

9. g) Government removal of user fees: (where applicable) ☐

9. h) Other (describe what other approaches are being used): ☐

If ***Other***, specify: ____________________

COMMENT

10. Comment: (by the surveyor) ____________________

____

____

____

____

Interviewer's Initials : ____________________ *Interviewer's signature* : ____________________

Date form completed:

Day		Month		Year			

First data entry (initials): ____________________ Second data entry (initials): ____________________

Project ID: A 6 6 0 0 7 Country: ☐☐☐☐☐☐☐☐☐☐☐☐

Center ID: ☐☐☐☐ Year of reported data: ☐☐☐☐ Month of reported data: ☐☐

SERVICES AND REFERRALS

1. Date of questionnaire: Day ☐☐ Month ☐☐ Year ☐☐☐☐

1.a. Are you completing the Baseline or the Endline? ☐

1 = Baseline **(complete Q4a - f and Q5a - f)** 2 = Endline **(complete Q4g - l and Q5g - l)**

2. Is the National family planning guidelines present in the facility? ☐

1 = No 2 = Yes

2a = If no, why not? _______

3. Are there any family planning check-lists and/or job-aids available in the facility? ☐

1 = No 2 = Yes

3a = If no, why not? _______

4. How many clients visited the facility for family planning counseling and services per month?

Only complete Q4a to Q4f for FPL Baseline (for the past six months)

	Date (MM/YYYY)	Number of clients
4. a) Month 1:	☐☐ ☐☐☐☐	☐☐☐
4. b) Month 2:	☐☐ ☐☐☐☐	☐☐☐
4. c) Month 3:	☐☐ ☐☐☐☐	☐☐☐
4. d) Month 4:	☐☐ ☐☐☐☐	☐☐☐
4. e) Month 5:	☐☐ ☐☐☐☐	☐☐☐
4. f) Month 6:	☐☐ ☐☐☐☐	☐☐☐

Only complete Q4g to Q4l for FPL Endline per month (for the past six months)

	Date (MM/YYYY)	Number of clients
4. g) Month 1:	☐☐ ☐☐☐☐	☐☐☐
4. h) Month 2:	☐☐ ☐☐☐☐	☐☐☐
4. i) Month 3:	☐☐ ☐☐☐☐	☐☐☐
4. j) Month 4:	☐☐ ☐☐☐☐	☐☐☐
4. k) Month 5:	☐☐ ☐☐☐☐	☐☐☐
4. l) Month 6:	☐☐ ☐☐☐☐	☐☐☐

Project ID: A 6 6 0 0 7 Country:

Center ID: Year of reported data: Month of reported data:

SERVICES AND REFERRALS (CONTINUED)

5. Any referrals for family planning services to other healthcare facilities per month?

1 = No (***Skip to Q8***) 2 = Yes

If ***Yes*** , how many referrals per month?

Only complete Q5a to Q5f for FPL Baseline per month (for the past six months)

	Date (MM/YYYY)	Number of referrals
5. a) Month 1:		
5. b) Month 2:		
5. c) Month 3:		
5. d) Month 4:		
5. e) Month 5:		
5. f) Month 6:		

Only complete Q5g to Q5l for FPL Endline per month (for the past six months)

	Date (MM/YYYY)	Number of referrals
5. g) Month 1:		
5. h) Month 2:		
5. i) Month 3:		
5. j) Month 4:		
5. k) Month 5:		
5. l) Month 6:		

Project ID: A 6 6 0 0 7 Country: ☐☐☐☐☐☐☐☐☐☐☐☐

Center ID: ☐☐☐☐ Year of reported data: ☐☐☐☐ Month of reported data: ☐☐

SERVICES AND REFERRALS (CONTINUED)

6. How many referrals in the last 6 months are due to the following reasons?

6. a) Contraceptive service not provided at the current site (***Goto Q7***): ☐☐☐☐

6. b) Adverse effect of a contraceptive method (**Goto Q8**): ☐☐☐☐

6. c) Other reason (**Specify and then Q8**): ☐☐☐☐

If ***Other reason*** , specify: ____________________

7. Number of referrals (due to contraceptive service not provided)?
00 if No referrals

7. a) Combined estrogen progesterone oral contraceptive pill: ☐☐

7. b) Progestin-only contraceptive pill: ☐☐

7. c) Combined estrogen progesterone injectable contraceptive (CIC): ☐☐

7. d) Progestin-only injectable contraceptive (DMPA or NET-EN): ☐☐

7. e) Male condom: ☐☐

7. f) Female condom: ☐☐

7. g) Emergency contraceptive pill: ☐☐

7. h) Cycle beads for standard days method: ☐☐

7. i) Vaginal ring: ☐☐

7. j) Male sterilization: ☐☐

7. k) Female sterilization: ☐☐

7. l) Implant (sub dermal implant): ☐☐

7. m) Copper IUD: ☐☐

7. n) Levonorgestrel Intrauterine Device: ☐☐

7. o) Other natural family planning method: ☐☐

7. p) Lactational Amenorrhea Method: ☐☐

7. q) Diaphragm: ☐☐

Project ID: A 6 6 0 0 7 Country: ☐☐☐☐☐☐☐☐☐☐☐☐

Center ID: ☐☐☐☐ Year of reported data: ☐☐☐☐ Month of reported data: ☐☐

INFRASTRUCTURE OF THE FACILITY

Please indicate for each question:

1 = No 2 = Yes

8. There are clear signs in the clinic on days and times in which services are available: ☐

9. The opening hours are convenient for clients, especially women and girls from key populations, including adolescents: ☐

10. There is a reception desk at the facility to help inform and guide clients: ☐

11. There is a separate room for FP/contraception services (e.g. for private examination, insertion/removal of IUDs, counselling room): ☐

12. a) There are separate waiting rooms, especially for adolescents: ☐

12. b) If **Yes**, how many? ☐☐

12. c) If No, where do adolescents wait? ______________

13. The counselling rooms are curtained-off from others to listen and hear: ☐

14. The examination rooms are curtained-off from others to listen and hear: ☐

15. There are simple seating and waiting areas for users sheltered from sun, wind and rain: ☐

16 There are written information and materials available on the various contraceptive methods, so that users can take materials home to read: ☐

17. There are recording systems in place to maintain accurate, confidential records on each user's in order to provide continuity of care and follow-up on:

17. a) Clients contact information: ☐

17. b) Relevant medical history of the client: ☐

17. c) History of contraceptive method use: ☐

18. There are posters about violence against women (e.g. that it is not acceptable, can cause harm/health problems, you can discuss with your provider) and/or leaflets available: ☐

Project ID: A 6 6 0 0 7 Country:

Center ID: Year of reported data: Month of reported data:

COMMODITIES

Please indicate for each question:

1 = No 2 = Yes 3 = Not applicable

19. a) Does this facility continue to stock contraceptive commodities at this site? ☐

If No, goto Q20

19.b) Are there any of the following stocks of reproductive health medicines and commodities available today in this facility?

1. Combined Estrogen Progesterone oral contraceptive pill: ☐
2. Progestin-only contraceptive pill: ☐
3. Combined estrogen progesterone injectable contraceptives (CIC): ☐
4. Progestin-only injectable contraceptive (DMPA or NET-EN): ☐
5. Male condom: ☐
6. Female condom: ☐
7. Emergency contraceptive pill: ☐
8. Cycle beads for standard days method: ☐
9. Vaginal ring: ☐
10. Implant (sub-dermal implant): ☐
11. Copper IUD: ☐
12. Levonorgestrel Intrauterine Device: ☐
13. Other natural family planning method: ☐
14. Diaphragm: ☐

20. Are the following contraceptive methods provided at the facility?

20. a) Male sterilization: ☐

20. b) Female sterilization: ☐

Project ID: A 6 6 0 0 7 Country: ☐☐☐☐☐☐☐☐☐☐☐☐

Center ID: ☐☐☐☐ Year of reported data: ☐☐☐☐ Month of reported data: ☐☐

HUMAN RESOURCES

21. Have the family planning service providers received any training in family planning in the last 6 months? ☐
    1 = No 2 = Yes

21 a. If no or yes, please provide details _______________

22. Have the family planning service providers received any training in adolescent sexual and reproductive health (including family planning) in the last 6 months? ☐
    1 = No 2 = Yes

22 a. If no or yes, please provide details _______________

23. Total number of human resources (staffing) in provision of family planning services in the past 2 months: ☐☐

24. Sex of Service providers available? ☐
    1 = Female 3 = Both
    2 = Male

25. Number of personnel cadres (directly involved in family planning care) available at the health facility:

    25. a) OB/Gyn specialist: ☐

    25. b) Medical doctor - General practitioner: ☐

    25. c) Nurse: ☐

    25. d) Midwife: ☐

    25. e) Other health workers (recognized and trained by government e.g. Social worker, Counselor...): ☐

    If ***Other*** , specify: ____________________

COMMENT

26. Comment: (by the surveyor) ____________________

____

Interviewer's Initials : ____________________ *Interviewer's signature* : ____________________

Date form completed:

Day		Month		Year			

First data entry (initials): ____________________ Second data entry (initials): ____________________

World Health Organization	A66007 - Health systems analysis and evaluations of the barriers to availability, utilization and readiness of sexual and reproductive health services in COVID-19 affected areas<br><br>Post Abortion Care module	ABO<br><br>Page 1/7<br><br>V 0.21 (6 Oct 2021)

Project ID: A 6 6 0 0 7 Country: [ ]

Center ID: [ ] Year of reported data: [ ] Month of reported data: [ ]

SERVICES AND REFERRALS

1. Date of questionnaire:

Day	Month	Year

1.a. Are you completing the Baseline or the Endline? [ ]

1 = Baseline **(complete Q4a - f and Q5a - f)** 2 = Endline **(complete Q4g - l and Q5g - l)**

2. Is the National abortion guidelines present in the facility? [ ]

1 = No 2 = Yes 3: Do not Know

2.a. If no, why not? ________

3. Are there any safe abortion check-lists and/or job-aids available in the facility? [ ]

1 = No 2 = Yes 3: Do not Know

3a = If no, why not? ________

4. How many clients visited the facility for safe abortion/post-abortion counseling and services per month?

Only complete Q4a to Q4f for ABO Baseline per month (for the past six months)

	Date (MM/YYYY)	Number of clients
4. a) Month 1:		
4. b) Month 2:		
4. c) Month 3:		
4. d) Month 4:		
4. e) Month 5:		
4. f) Month 6:		

Only complete Q4g to Q4l for Endline per month (for the past six months)

	Date (MM/YYYY)	Number of clients
4. g) Month 1:		
4. h) Month 2:		
4. i) Month 3:		
4. j) Month 4:		
4. k) Month 5:		
4. l) Month 6:		

Project ID: A 6 6 0 0 7 Country: [ ]

Center ID: [ ] Year of reported data: [ ] Month of reported data: [ ]

SERVICES AND REFERRALS (CONTINUED)

5) Any referrals for abortion services to other healthcare facilities per month? [ ]

1 = No (***Skip to Q8***) 2 = Yes

If ***Yes*** , how many referrals per ***month*** ?

Only complete Q5a to Q5f for Baseline per month (for the past six months)

	Date (MM/YYYY)	Number of referrals
5. a) Month 1:		
5. b) Month 2:		
5. c) Month 3:		
5. d) Month 4:		
5. e) Month 5:		
5. f)Month 6:		

Only complete Q5g to Q5l for Endline per month (for the past six months)

	Date (MM/YYYY)	Number of referrals
5. g) Month 1:		
5. h) Month 2:		
5. i) Month 3:		
5. j) Month 4:		
5. k) Month 5:		
5. l) Month 6:		

6. What is the major reason of the referrals? **If all the answers (Q6a to Q6c) are NO, please go to Q8**

1 = No 2 = Yes

6. a) Lack or absence of specific procedures to treat women seeking postabortion services [ ]

6. b) Lack or absence of Medical Termination (pharmacological and/or surgical) of the pregnancy [ ]

6. c) Other reason [ ]

If ***Other reason*** , specify: ______________________

Project ID: A 6 6 0 0 7 Country:

Center ID: Year of reported data: Month of reported data:

SERVICES AND REFERRALS (CONTINUED)

7. Number of referrals per type of abortion care?

00 if No referral

7. a) Missed Abortion:

7. b) Treatment of incomplete abortion:

7. c) Treatment of uterine perforation:

7. d) Treatment of abortion−related sepsis/ infection:

7. e)Treatment of abortion−genital trauma:

7. f) Treatment of abortion−related hemorrhage/ bleeding:

7. g) Other complication:

If ***Other complication*** , specify: ______________________

INFRASTRUCTURE OF THE FACILITY

Please indicate for each question:

1 = No 2 = Yes

8. There are clear signs in the clinic on days and times in which services are available:

9. The opening hours are convenient for clients, especially women and girls from key populations, including adolescents:

10. There is a reception desk at the facility to help inform and guide clients:

11. For adult clients: There is a separate room for abortion services for clients (e.g. for private examination):

1 = No 2 = Yes

12. a) For Adolescents: There are separate waiting rooms:

12. b) If ***Yes*** , how many?

12. c) If No, where do adolescents wait? --

13. The counselling rooms are separate for private and confidential consultation:

14. The examination rooms are separate for private and confidential consultation:

15. There are simple seating and waiting areas (e.g. for users sheltered from sun, wind and rain):

16. There are written information and materials available on the various safe abortion methods, so that users can take materials home to read:

16 a. There are posters about violence against women (e.g. that it is not acceptable, can cause harm/health problems, you can discuss with your provider) and/or leaflets available:

Project ID: A 6 6 0 0 7 Country:

Center ID: Year of reported data: Month of reported data:

INFRASTRUCTURE (CONTINUED)

17. There are recording systems in place to maintain accurate, confidential records on each user in order to provide continuing care and follow-up:

1 = No 2 = Yes

17. a) Clients contact information:

17. b) Relevant medical history of the client:

17. c) History of abortion service use:

COMMODITIES

18. Does the facility continue to stock abortion commodities?

1 = No 2 = Yes

19. Are there any of the following stocks of medical devices available today in this facility?

1 = No 2 = Yes 3 = Not applicable

19. a) 22 gauge spinal needles for paracervical block:

19. b) 21 gauge needles for drug administration:

19. c) Syringes 5 ml:

19. d) Syringes 10 ml:

19. e) Syringes 20 ml:

19. f) IV (intravenous) line:

19. g) Blood pressure equipment:

19. h) Stethoscope:

19. i) Speculum:

19. j) Tenaculum:

19. k) Tapered dilators up to 51 mm or equivalent circumference:

19. l) Electric vacuum aspirator (with 14 or 16 mm cannulae):

19. m) MVA aspirator and cannulae up to 12 mm:

19. n) Uterine evacuation forceps:

Project ID: A 6 6 0 0 7 Country: ______

Center ID: ______ Year of reported data: ______ Month of reported data: ______

COMMODITIES (CONTINUED)

19. o) Large, postpartum flexible curette: ☐

19. p) Stainless steel bowl for preparing solution: ☐

19. q) Instrument tray: ☐

19. r) Clear glass dish for tissue inspection: ☐

19. s) Oxygen and Ambu bag: ☐

19. t) On-site access to an ultrasound machine (optional in some settings): ☐

19. u) Long needle-driver and suture: ☐

19. v) Scissors: ☐

19. w) Uterine packing: ☐

19. x) Blood bank: ☐

20. Any of the following medicines were out of stock in this facility during the last 6 months?

1 = No 2 = Yes 3 = Not applicable

20. a) Misoprostol: ☐

20. b) Osmotic dilators: ☐

20. c) Mifepristone: ☐

20. d) Analgesics: ☐

20. e) Anxiolytics: ☐

If ***Yes*** , specify anxiolytics: ______________________ ☐☐☐ - ☐ **ICD10**

20. f) Antibiotics: ☐

If ***Yes*** , specify antibiotics: ______________________ ☐☐☐ - ☐ **ICD10**

20. g) Fluids (saline, sodium lactate, glucose): ☐

20. h) Lidocaine for paracervical block: ☐

20. i) Appropriate antagonists to medications used for pain: ☐

20. j) Uterotonics (oxytocin, misoprostol or ergometrine): ☐

20. k) Antiseptic solution (non-alcohol based) to prepare the cervix: ☐

20. l) Sterilization or high-level disinfection solutions and materials: ☐

Project ID: A 6 6 0 0 7 Country:

Center ID: Year of reported data: Month of reported data:

COMMODITIES (CONTINUED)

21. Any of the following sundries and services were lacking in this facility during the last 6 months?

1 = No 2 = Yes 3 = Not applicable

21. a) Clean examination gloves: ☐

21. b) Gown: ☐

21. c) Face protection: ☐

21. d) Clean water: ☐

21. e) Detergent or soap: ☐

21. f) Instrument soaking solution: ☐

21. g) Gauze sponges or cotton balls: ☐

21. h) Sanitary napkins or cotton wool: ☐

21. i)Strainer (metal, glass or gauze): ☐

21. j) Adequate toilet facilities: ☐

21. k) Printed information for clients on post-procedure self-care: ☐

21. l) Clear referral mechanisms to higher-level facility, when needed: ☐

21. m) Private area with chairs separate from antenatal or labour care, for women who wait in clinic for expulsion: ☐

HUMAN RESOURCES

22. Have the abortion service providers received any training in safe abortion services in the last 6 months? ☐

1 = No 2 = Yes

23. Have the abortion service providers received any training in adolescent sexual and reproductive health (including family planning) in the last 6 months? ☐

1 = No 2 = Yes

24. Total number of human resources (staffing) in provision of safe abortion services in the past 2 months: ☐☐

25. Gender of service providers available? ☐

1 = Female only 3 = Both

2 = Male only

	A66007 - Health systems analysis and evaluations of the barriers to availability, utilization and readiness of sexual and reproductive health services in COVID-19 affected areas	ABO
World Health Organization	Post Abortion Care module	Page 7/7<br>V 0.21 (6 Oct 2021)

Project ID: A 6 6 0 0 7 Country:

Center ID: Year of reported data: Month of reported data:

HUMAN RESOURCES

26. Type of personnel (directly involved in safe abortion care) available at the health facility:

1 = No 2 = Yes

26. a) OB/Gyn specialist: ☐

26. b) Medical doctor/ general practitioner: ☐

26. c) Nurse: ☐

26. d) Midwife: ☐

26. e) Other health workers (recognized and trained by government e.g. Social worker, Counselor...): ☐

If *Other* , specify: ____________________

COMMENT

27. Comment: ____________________

____

____

____

____

Interviewer's Initials : ____________________ *Interviewer's signature* : ____________________

Date form completed:

Day		Month		Year			

First data entry (initials): ____________________ Second data entry (initials): ____________________

Project ID: A 6 6 0 0 7 Country: ☐☐☐☐☐☐☐☐☐☐☐☐

Center ID: ☐☐☐☐ Year of reported data: ☐☐☐☐ Month of reported data: ☐☐

HIV/AIDS

1. Does this facility offer HIV counselling and testing services? ☐
   1 = No *2 = Yes*

2. Do you have the national HIV counselling and testing guidelines available in this facility today? ☐
   1 = No *2 = Yes*

2a = If no, why not? _______

3. Does this facility have HIV rapid test kits (with valid expiration date) in stock today, ready for client testing? ☐
   1 = No *2 = Yes*

4. Does this facility have condoms available in this service site today to give to clients receiving services? ☐
   1 = No *2 = Yes*

5. Does this facility offer HIV & AIDS antiretroviral prescription or antiretroviral treatment follow-up services? ☐
   1 = No *2 = Yes*

STIs component

6. Does this facility offer diagnosis or treatment of STIs (other than HIV)?services? ☐
   1 = No *2 = Yes*

7. Do providers in this facility diagnose STIs? ☐
   1 = No *2 = Yes*

8. Do providers in this facility prescribe treatment for STIs? ☐
   1 = No *2 = Yes*

9. Do you have the national guidelines for the diagnosis and treatment of STIs available in this facility today? ☐
   1 = No *2 = Yes*

10. Have you or any provider(s) of STI services received any training in STI diagnosis and treatment in the last two years? ☐
    1 = No *2 = Yes*

Project ID: A 6 6 0 0 7 Country: ☐☐☐☐☐☐☐☐☐☐☐☐

Center ID: ☐☐☐☐ Year of reported data: ☐☐☐☐ Month of reported data: ☐☐

STIs component (CONTINUED)

11. If you offer diagnosis or treatment of STIs (other than HIV) what is the target population/s??

1= Male adolescents
2= Female adolescents
3= Gay man/Men who have sex with Men (MSM)
4= Transgender women (TGW)
5= Sex workers (both male and female)
6= Person living with HIV (PLWH)
7= Adults (both men and women)

[check all that apply]

	1	2	3	4	5	6	7
11a) Chlamydia trachomatis	☐	☐	☐	☐	☐	☐	☐
11b) Neisseria gonorrhoeae	☐	☐	☐	☐	☐	☐	☐
11c) Syphilis	☐	☐	☐	☐	☐	☐	☐
11d) Trichomonas Vaginalis	☐	☐	☐	☐	☐	☐	☐
11e) Bacterial Vaginosis	☐	☐	☐	☐	☐	☐	☐
11f) Mycoplasma genitalium	☐	☐	☐	☐	☐	☐	☐

HIV/STIs testing component

12. Does this facility offer any of the following tests on-site? ☐

1 = No *2 = Yes*

[If Q12 = Yes, check all that apply]

- 12a) Syphillis Rapid Testing ☐
- 12b) HIV rapid testing ☐
- 12c) Syphilis dark field microscopy ☐
- 12d) Urine rapid tests for pregnancy ☐
- 12e) Urine protein dipstick testing ☐
- 12f) Urine glucose dipstick testing ☐
- 12g) Urine ketone dipstick testing ☐
- 12h) Dry Blood Spot (DBS) collection for HIV viral load or EID ☐

STIs vaccines component

13. Does this facility or other public healthcare facilities in your area offer immunization services? ☐

1 = No **if "No"go to Q16** *2 = Yes*

14. Are there vaccinations available for the following sexually transmitted pathogens

1 = No *2 = Yes*

- 14. a) HAV ☐
- 14. b) HBV ☐
- 14. c) HPV ☐

Project ID: A 6 6 0 0 7 Country:

Center ID: Year of reported data: Month of reported data:

STIs vaccines component (CONTINUED)

15. Is the HIV and STIs testing and counselling service room or area a private room/area with auditory and visual privacy?
1 = No *2 = Yes*

Partner notification (PN) and Contact tracing (CT) component

16. If Q14="Yes", what is the target population?
1 = No *2 = Yes*
16a) Male adolescents
16b) Female adolescents
16c) Gay man/Men who have sex with Men (MSM)
16d) Transgender women (TGW)
16e) Sex workers (both male and female)
16f) Person living with HIV (PLWH)
16g) Adults (both men and women)

17. Do you have guidelines for partner notification and contact tracing available?
1 = No *2 = Yes*
17a) Partner Notification (PN)
17ai) (for HIV) yes, national guidelines
17aii) (for HIV) yes, local guidelines
17aiii) (for other STIs) yes, national guidelines
17aiv) (for other STIs) yes, local guidelines
17b) Contact Tracing (CT)
17bi) (for HIV) yes, national guidelines
17bii) (for HIV) yes, local guidelines
17biii) (for other STIs) yes, national guidelines
17biv) (for other STIs) yes, local guidelines

18. Are any of the following drugs for STIs other than HIV available today in this facility?
1 = No 2 = Yes *[check all that apply]*
18a) Doxycyclin
18b) Azithromycin
18c) Penicillin
18d) Erythromycin
18e) Amoxicillin
18f) Methronidazole
18g) Ceftriaxone
18h) Cefixime
18i) Spectinomycin

Project ID: A 6 6 0 0 7 Country: ☐

Center ID: ☐ Year of reported data: ☐ Month of reported data: ☐

Partner notification (PN) and Contact tracing (CT) component (CONTINUED)

19. How many clients visited the facility for STI counseling and services per month?

19.a1. Plase indicate if you are completing the Baseline or the Endline? ☐

1 = Baseline **(complete Q19a - f)** 2 = Endline **(complete Q19g - l)**

Only complete Q19a to Q19f for STI Baseline per month (for the past six months)

	Date (MM/YYYY)	Number of clients
19. a) Month 1:	☐☐ ☐☐☐☐	☐☐☐
19. b) Month 2:	☐☐ ☐☐☐☐	☐☐☐
19. c) Month 3:	☐☐ ☐☐☐☐	☐☐☐
19. d) Month 4:	☐☐ ☐☐☐☐	☐☐☐
19. e) Month 5:	☐☐ ☐☐☐☐	☐☐☐
19. f) Month 6:	☐☐ ☐☐☐☐	☐☐☐

Only complete Q19g to Q19l for Endline per month (for the past six months)

	Date (MM/YYYY)	Number of clients
19. g) Month 1:	☐☐ ☐☐☐☐	☐☐☐
19. h) Month 2:	☐☐ ☐☐☐☐	☐☐☐
19. i) Month 3:	☐☐ ☐☐☐☐	☐☐☐
19. j) Month 4:	☐☐ ☐☐☐☐	☐☐☐
19. k) Month 5:	☐☐ ☐☐☐☐	☐☐☐
19. l) Month 6:	☐☐ ☐☐☐☐	☐☐☐

Project ID: A 6 6 0 0 7

Country:

Center ID:

Year of reported data:

Month of reported data:

VIOLENCE AGAINST WOMEN SERVICES AND REFERRALS

1. Date of questionnaire: Day / Month / Year

1.a. Are you completing the Baseline or the Endline?

1 = Baseline **(complete Q4a - f)** 2 = Endline **(complete Q4g - l)**

2. Are the National guidelines for provision of health care to women subjected to domestic and/or sexual violence available in the facility?

1 = No 2 = Yes 3. Do not know

2. a) Domestic violence:

2. b) Sexual violence:

2a = If no, why not? _______

3. Are there any domestic and/or sexual violence check-lists and/or job-aids available in the facility?

1 = No 2 = Yes 3. Do not know

3. a) Domestic violence:

3. b) Sexual violence:

3a = If no, why not? _______

4. How many clients visited the facility for domestic and/or sexual violence counseling and services per month?

Only complete Q4a to Q4f for GBV Baseline per month (for the past six months)

	Date (MM/YYYY)	Number of clients
4. a) Month 1:		
4. b) Month 2:		
4. c) Month 3:		
4. d) Month 4:		
4. e) Month 5:		
4. f) Month 6:		

Only complete Q4g to Q4l for GBV Endline per month (for the past six months)

	Date (MM/YYYY)	Number of clients
4. g) Month 1:		
4. h) Month 2:		
4. i) Month 3:		
4. j) Month 4:		
4. k) Month 5:		
4. l) Month 6:		

Project ID: A 6 6 0 0 7 Country:

Center ID: Year of reported data: Month of reported data:

VIOLENCE AGAINST WOMEN SERVICES AND REFERRALS (CONTINUED)

5. Does the facility provide the following aspects of care/management of domestic violence?

1 = No 2 = Yes

5. a) Ask/identify cases of domestic violence: ☐

5. b) Offer injury or other medical care to those who need it: ☐

5. c) Offer psychological support/crisis counselling/first-line support to those who disclose (internal referral): ☐

5. d) Refer to NGOs, counselling, or any other services outside the health facility that the woman might need: ☐

6. Does the facility provide the following elements of post-rape care/care to survivors of sexual violence?

1 = No 2 = Yes

6. a) Injury treatment: ☐

6. b) Emergency contraception: ☐

6. c) HIV post-exposure prophylaxis: ☐

6. d) STI prophylaxis/presumptive treatment: ☐

6. e) Medical abortion to those who get pregnant: ☐

6. f) Surgical abortion to those who get pregnant: ☐

6. g) ANC and delivery care to those who get pregnant: ☐

6. h) Psychological support/crisis counselling/first line-support: ☐

6. i) Referral to mental health or specialized VAW services outside the health facility: ☐

7. If you answered yes to 6d or 6i, Where are cases of sexual assault and partner violence referred to?

1 = No 2 = Yes

7. a) Police: ☐

7. b) Protection services: ☐

7. c) Shelter services: ☐

7. d) Women's NGOs: ☐

7. e) Crisis counseling services: ☐

7. f) Livelihood support: ☐

7. g) Legal services: ☐

Project ID: A 6 6 0 0 7 Country: ____________

Center ID: ____ Year of reported data: ____ Month of reported data: __

VIOLENCE AGAINST WOMEN SERVICES AND REFERRALS (CONTINUED)

8. Do you have a referral directory with names and contact details of organizations/services that respond to cases of violence? ☐
   1 = No 2 = Yes
   8. a) If yes, was it updated within the last year? ☐

INFRASTRUCTURE OF THE FACILITY

9. There are clear signs in the clinic on days and times in which services are available: ☐
   1 = No 2 = Yes

10. The counselling rooms are separate and available for private and confidential consultation: ☐
   1 = No 2 = Yes

11. The examination rooms are separate and available for private and confidential consultation: ☐
   1 = No 2 = Yes

12. There are visible posters about violence against women (e.g. that it is not acceptable, can cause harm/health problems, you can discuss with your provider) and/or leaflets available: ☐
   1 = No 2 = Yes

13. Does the health management information system include means to record details of:
   1 = No 2 = Yes

   13. a) Sexual assault: ☐
   13. b) Intimate partner/domestic violence: ☐

14. Does the facility use any of the following to maintain confidentiality of the documentation of cases of violence?
   1 = No 2 = Yes

   14. a) The facility does not have any method for maintaining confidentiality of documentation of violence ☐

   14. b) Identifying information is removed or kept separate from incident record ☐

   14. c) Records, registers and forms are kept in a secure storage with lock and pre-determined access ☐

   14. d) Electronic medical records are password protected with pre-determined access for those who need to care for survivors ☐

   14. e) Any take home cards or information does not have any direct indication of the survivor's abuse ☐

   14. f) Other (specify): ______________________________ ☐

Project ID: A 6 6 0 0 7 Country: ☐☐☐☐☐☐☐☐☐☐☐☐

Center ID: ☐☐☐☐ Year of reported data: ☐☐☐☐ Month of reported data: ☐☐

COMMODITIES

15. Does the facility have the following essential supplies and equipment for post-rape care today during this visit?

Please indicate for each question:

1 = No 2 = Yes

15. a) Emergency contraception: ☐

15. b) Antiretroviral drugs for post-exposure prophylaxis for HIV prevention: ☐

15. c) Antibiotics/other drugs for treatment or prophylaxis for sexually transmitted infection: ☐

15. d) Hepatitis B vaccination: ☐

15. e) Job aids (for example, flow charts, algorithms, pictograms): ☐

15. f) Documentation forms (for example, medical intake forms, police forms for forensic evidence, medico-kegal certificates, referral forms): ☐

15. g) Communication materials about violence against women: ☐

15. h) Sanitary pads: ☐

15. i) Examination table/couch (with curtains or screens if needed for privacy): ☐

15. j) Secure record storage cabinets with a lock: ☐

15. k) Adequate light source (lamp or torch): ☐

15. l) Speculum: ☐

15. m) Pregnancy testing kits: ☐

15. n) Rapid tests for HIV/syphilis: ☐

15. o) Urinalysis kits: ☐

15. p) Test strips for vaginal infections: ☐

15. q) Supplies for wound care: ☐

15. r) Analgesics: ☐

15. s) Anti-emetics: ☐

15. t) Tetanus toxoid: ☐

15. u) Consent forms: ☐

15. v) Sheets, blankets, and towels: ☐

Project ID: A 6 6 0 0 7 Country:

Center ID: Year of reported data: Month of reported data:

HUMAN RESOURCES

16. Have health care providers received training on responding to intimate partner/domestic violence in the last 6 months?
    1 = No (***If No go to Q.18***) 2 = Yes

17. If Yes, how many health care providers in each cadre received training on intimate partner/domestic violence in the last 6 months?

    17. a) Medical doctor - General practitioner:

    17. b) OB/Gyn specialist:

    17. c) Nurse:

    17. d) Midwife:

    17. e) Other health workers (recognized and trained by government e.g. Social worker, Counselor...):
    If ***Other*** , specify: ____________

    17.f) Gender
    1 = Female 3 = Both
    2 = Male

18. Have health care providers received training on responding to sexual violence in the last 6 months?
    1 = No (***Goto Q20***) 2 = Yes

19. If ***Yes*** , how many health care providers in each cadre received training on sexual violence In the last 6 months?

    19. a) Medical doctor - General practitioner:

    19. b) OB/Gyn specialist:

    19. c) Nurse:

    19. d) Midwife:

    19. e) Other health workers (recognized and trained by government e.g. Social worker, Counselor...)
    If ***Other*** , specify: ____________

COMMENT

20. Comment: ____________

Interviewer's Initials : ____________ *Interviewer's signature* : ____________

Date form completed:

Day	Month	Year
