# Supplementary material for: Family planning and abortion service availability and utilisation during the COVID-19 pandemic in Ghana
Source: Reprod Health. 2025 Nov 20;22(Suppl 3):234. doi: 10.1186/s12978-025-02122-x (PMC12632033; doi:10.1186/s12978-025-02122-x)
Supplement: Supplementary file 2 — Supplementary Material 2 [file 12978_2025_2122_MOESM2_ESM.docx]

Additional file 2 Survey Respondent characteristics

| **Characteristic** | **Frequency** | **Percentage** | |
| --- | --- | --- | --- |
| **Age** | 30.6 + 6.7 |  | |
| **School years** | 10.2 + 3.9 |  | |
| **Marital Status** |  |  | |
| Single | 328 | 33.1 | |
| Cohabiting | 646 | 65.3 | |
| Separated | 5 | 0.5 | |
| Divorced | 4 | 0.4 | |
| Widowed | 7 | 0.7 | |
| **Tested COVID since 2019** |  |  | |
| Yes | 116 | 11.7 | |
|  |  |  | |
| **PCR Test (n=115)** |  |  | |
| Not Done | 55 | 47.8 | |
| Negative | 54 | 47.0 | |
| Positive | 6 | 5.2 | |
| **Currently Pregnant (n=107)** |  |  | |
| Yes | 68 | 6.9 | |
| **Gestational Age** |  |  |  |
| 1St Trimester | 53 | 49.5 |  |
| 2Nd Trimester | 25 | 23.4 |  |
| 3Rd Trimester | 29 | 27.1 |  |
|  |  |  | |
