# Supplementary material for: Family planning and abortion service availability and utilisation during the COVID-19 pandemic in Ghana
Source: Reprod Health. 2025 Nov 20;22(Suppl 3):234. doi: 10.1186/s12978-025-02122-x (PMC12632033; doi:10.1186/s12978-025-02122-x)
Supplement: Supplementary file 3 — Supplementary Material 3 [file 12978_2025_2122_MOESM3_ESM.docx]

Additional file 3 Characteristics of Health Care Providers

| **Variable** | **Frequency (N = 16)** |
| --- | --- |
| **Sex** |  |
| Male | 3 |
| Female | 13 |
| **Age range (min-max yrs)** | 31 – 58 |
| Mean Age | 43.6 |
| **Ethnicity** |  |
| Akan | 8 |
| Ga-Dangme | 3 |
| Ewe | 4 |
| Not provided | 1 |
| **Marital Status** |  |
| Married | 16 |
| **Educational status** |  |
| SHS | 1 |
| Tertiary | 15 |
| **Religion** |  |
| Christianity | 16 |
| **Rank** |  |
| Deputy Director of Nursing | 1 |
| Health Assistant | 1 |
| Medical Superintendent | 4 |
| Midwife Officer | 1 |
| not provided | 1 |
| Nursing officer | 2 |
| Principal Nursing Officer | 2 |
| Senior Nursing Officer | 3 |
| Senior Staff Nurse | 1 |
