# Supplementary material for: Family planning and abortion service availability and utilisation during the COVID-19 pandemic in Ghana
Source: Reprod Health. 2025 Nov 20;22(Suppl 3):234. doi: 10.1186/s12978-025-02122-x (PMC12632033; doi:10.1186/s12978-025-02122-x)
Supplement: Supplementary file 4 — Supplementary Material 4 [file 12978_2025_2122_MOESM4_ESM.docx]

Additional file 4 Characteristics of participants for In-depth Interviews (IDIs)

| Variable | Frequency (N=66) |
| --- | --- |
| **Sex** |  |
| Male | 30 |
| Female | 36 |
| **Age (min-max years)** | 18 – 49 |
| **Mean Age** | 33.2 |
| **Marital Status** |  |
| Single | 23 |
| Cohabiting | 6 |
| Married | 36 |
| Divorced | 1 |
| **Educational status** |  |
| No formal education | 3 |
| Primary | 4 |
| JHS/ SHS/ Certificate | 38 |
| SHS | 15 |
| Tertiary | 1 |
| Not provided | 5 |
| **Occupation** |  |
| Trading/ business | 17 |
| White color jobs | 14 |
| Artisans/skilled labour* | 23 |
| Secretarial | 5 |
| Other unskilled labourª | 4 |
| Student/ unemployed | 3 |
| **Religion** |  |
| Christianity | 58 |
| Islam | 8 |
| **Residence** |  |
| Rented | 41 |
| Self-Owned | 12 |
| Family/ Relative’s house | 4 |
| Not provided | 9 |
| **Housing material/condition** |  |
| Cement/Block building | 14 |
| Wooden Kiosk | 2 |
| Not stated | 49 |
| Uncompleted house | 1 |
| **No. of pregnancies** |  |
| None | 3 |
| One - Two | 12 |
| Three - four | 18 |
| >= 5 | 3 |
| **No. of living children** |  |
| 0 | 10 |
| 1 | 13 |
| 2 | 12 |
| 3 | 12 |
| 4 | 5 |
| Not provided | 14 |
| *Includes seamstresses, carpenters, beauticians, etc.  a. Includes fuel attendants, cleaners, laborers, etc | |
