# Supplementary material for: Family planning and abortion service availability and utilisation during the COVID-19 pandemic in Ghana
Source: Reprod Health. 2025 Nov 20;22(Suppl 3):234. doi: 10.1186/s12978-025-02122-x (PMC12632033; doi:10.1186/s12978-025-02122-x)
Supplement: Supplementary file 5 — Supplementary Material 5 [file 12978_2025_2122_MOESM5_ESM.docx]

Additional file 5 Characteristics of participants for Focus Group Discussions

| Variable | Frequency (N=68) |
| --- | --- |
| **Sex** |  |
| Male | 17 |
| Female | 51 |
| **Age (min-max years)** | 16 – 56 |
| **Mean Age** | 32 |
| **Educational status** |  |
| No formal education | 2 |
| Primary | 6 |
| JHS/ SHS/ Certificate | 49 |
| Tertiary | 9 |
| Not provided |  |
| **Occupation** |  |
| Trading/ business | 20 |
| White color jobs | 6 |
| Artisans/skilled labour* | 11 |
| Other unskilled labourª | 10 |
| Student/ unemployed | 19 |
| **Residence** |  |
| Rented | 14 |
| Owned | 4 |
| Family/ Relative’s house | 1 |
| Not provided | 49 |
| **Age of Youngest Child** |  |
| <= 1 year | 6 |
| 1. to 7 years | 4 |
| >7 years | 1 |
| No child | 6 |
| Not provided | 51 |
| **No. of pregnancies** |  |
| None | 22 |
| One - Two | 24 |
| Three - four | 14 |
| >= 5 | 8 |
| **No. of living children** |  |
| 0 | 23 |
| 1 | 10 |
| 2 | 14 |
| 3 | 13 |
| >=4 | 8 |
| *Includes seamstresses, carpenters, beauticians, etc.  a. Includes fuel attendants, cleaners, laborers, etc | |
